# Push–Pull Derivatives Based on 2,4′-Biphenylene Linker with Quinoxaline, [1,2,5]Oxadiazolo[3,4-*B*]Pyrazine and [1,2,5]Thiadiazolo[3,4-*B*]Pyrazine Electron Withdrawing Parts

**DOI:** 10.3390/molecules27134250

**Published:** 2022-06-30

**Authors:** Egor V. Verbitskiy, Pascal le Poul, Filip Bureš, Sylvain Achelle, Alberto Barsella, Yuriy A. Kvashnin, Gennady L. Rusinov, Valery N. Charushin

**Affiliations:** 1 I. Postovsky Institute of Organic Synthesis, Ural Branch of the Russian Academy of Sciences, S. Kovalevskaya Str., 22, 620108 Ekaterinburg, Russia; kvashnin@ios.uran.ru (Y.A.K.); rusinov@ios.uran.ru (G.L.R.); valery-charushin-562@yandex.ru (V.N.C.); 2 Department of Organic and Biomolecular Chemistry, Chemical Engineering Institute, Ural Federal University, Mira St. 19, 620002 Ekaterinburg, Russia; 3 University Rennes, CNRS, Institut des Sciences Chimiques de Rennes—UMR 6226, 35000 Rennes, France; pascal.le-poul@univ-rennes1.fr; 4 Institute of Organic Chemistry and Technology, Faculty of Chemical Technology, University of Pardubice Studenská 573, 53210 Pardubice, Czech Republic; 5 Département d’Optique Ultrarapide et Nanophotonique, IPCMS, UMR CNRS 7504, Université de Strasbourg, 23 rue du Loess, BP 43, CEDEX 2, 67034 Strasbourg, France; alberto.barsella@ipcms.unistra.fr

**Keywords:** furazanopyrazine, [1,2,5]thiadiazolo[3,4-*b*]pyrazine, quinoxaline, donor–acceptor systems, twisted intramolecular charge transfer

## Abstract

A series of novel V-shaped quinoxaline, [1,2,5]oxadiazolo[3,4-*b*]pyrazine and [1,2,5]thiadiazolo[3,4-*b*]pyrazine push–pull derivatives with 2,4′-biphenylene linker were designed and their electrochemical, photophysical and nonlinear optical properties were investigated. [1,2,5]Oxadiazolo[3,4-*b*]pyrazine is the stronger electron-withdrawing fragment as shown by electrochemical, and photophysical data. All compounds are emissive in a solid-state (from the cyan to red region of the spectrum) and quinoxaline derivatives are emissions in DCM solution. It has been found that quinoxaline derivatives demonstrate important solvatochromism and extra-large Stokes shifts, characteristic of twisted intramolecular charge transfer excited state as well as aggregation induced emission. The experimental conclusions have been justified by theoretical (TD-)DFT calculations.

## 1. Introduction

**Figure 1:**
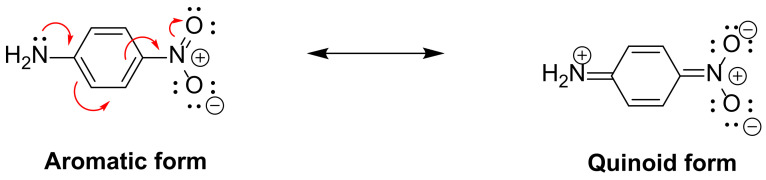
Limiting resonance forms of *p*-nitroaniline, a typical push–pull structure.

Push–pull derivatives with a D-π-A structure, where A and D are electron-withdrawing and electron-donating groups and the π represents a π-conjugated system, have been subject to a huge interest in the last decades due to their exceptional linear and nonlinear optical properties [1,2]. The D-A interaction into push–pull structures, also called intramolecular charge transfer (ICT), leads to the formation of low energy molecular orbitals whose electrons can be excited by visible light inducing colored materials [3,4,5,6]. The charge transfer absorption band will be red-shifted with an increase in the ICT strength. The limiting resonance forms of nitroaniline, a typical push–pull structure, are represented in Figure 1.

Push–pull molecular materials have found applications as fluorescent sensors [7,8,9,10], dye-sensitized solar cells (DSSC) [11,12,13,14], nonfullerene organic photovoltaic (OPV) materials [15,16,17], and numerous other optoelectronic devices [18,19,20,21]. The intramolecular charge transfer (ICT) in push–pull molecular materials can be modulated either by playing on the A/D couple [1,2,22,23] or by changing the nature and length of the π-conjugated bridge [1,2,24,25]. In this context, π-deficient heterocycles are particularly interesting as **A** part, as follows: the electron-lone pairs of heteroatoms of the heterocyclic core can be indeed used for protonation, complexation, alkylation, or the formation of a hydrogen bond, modifying their electron-withdrawing character and tuning the optical properties of corresponding push–pull structures by redistribution of charge density [26,27,28,29,30,31].

**Figure 2:**
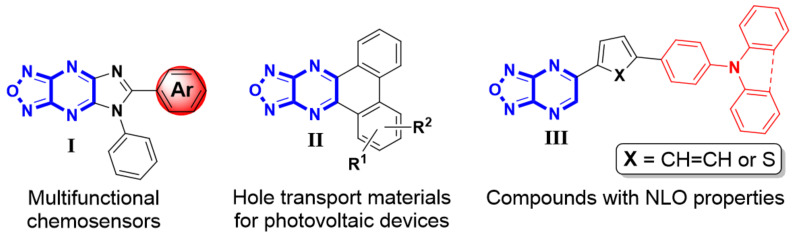
Representative compounds bearing furazanopyrazine scaffold.

1,2,5-Chalcogenadiazoles and their annulated derivatives have received great attention due to the remarkable combination of their interesting properties, synthesis, and applications in materials sciences [32,33,34,35]. Recently, some of us designed and demonstrated the possible applications of [1,2,5]oxadiazolo[3,4-*b*]pyrazine derivatives (also called furazanopyrazines) as effective multifunctional chemosensors (**I**) [36,37], charge-transport materials for photovoltaics (**II**) [38], and compounds with advanced nonlinear optical (NLO) properties (**III**) (Figure 2) [39].

Phenylene bridges are typical linkers in push–pull structures, even if the aromaticity of the π-linker is directed toward the ICT [40,41]. The 1,4-phenylene unit is generally used, but the *ortho* arrangement can be interesting due to the steric hindrance that can lead to a twisted intramolecular charge transfer (TICT) excited state with large Stokes shifts [42,43].

This article is a further extension of our recent studies that are focused on the design of novel chromophores based on strong electron-withdrawing [1,2,5]oxadiazolo[3,4-*b*]pyrazine fragments [39]. Herein we report the synthesis of a new series of V-shaped D–π–A push–pull systems based on [1,2,5]oxadiazolo[3,4-*b*]pyrazine, [1,2,5]thiadiazolo[3,4-*b*]pyrazine, and quinoxaline acceptor cores and 2,4′-biphenylene π-linker. The systematic experimental investigation of their electronic and photophysical properties has been carried out, and these results were rationalized with the help of theoretical (TD)-DFT calculation.

## 2. Results and Discussion

### 2.1. Synthesis

**Scheme 1:**
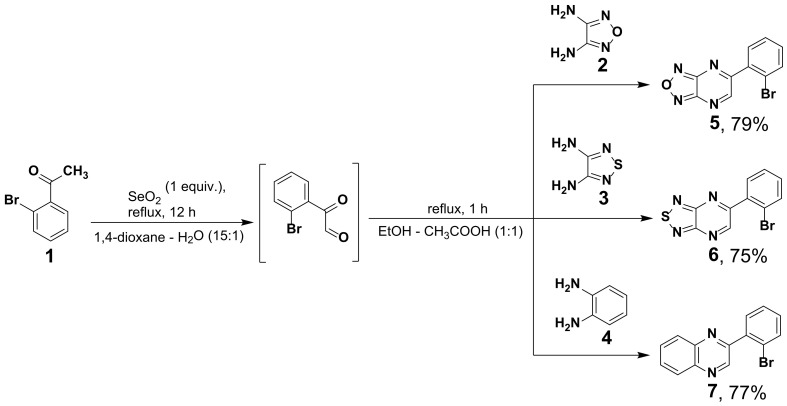
Synthesis of 5-(2-bromophenyl)-[1,2,5]oxadiazolo[3,4-*b*]pyrazine (**5**), 5-(2-bromophenyl)-[1,2,5]thiadiazolo[3,4-*b*]pyrazine (**6**), and 2-(2-bromophenyl)quinoxaline (**7**).

**Scheme 2:**
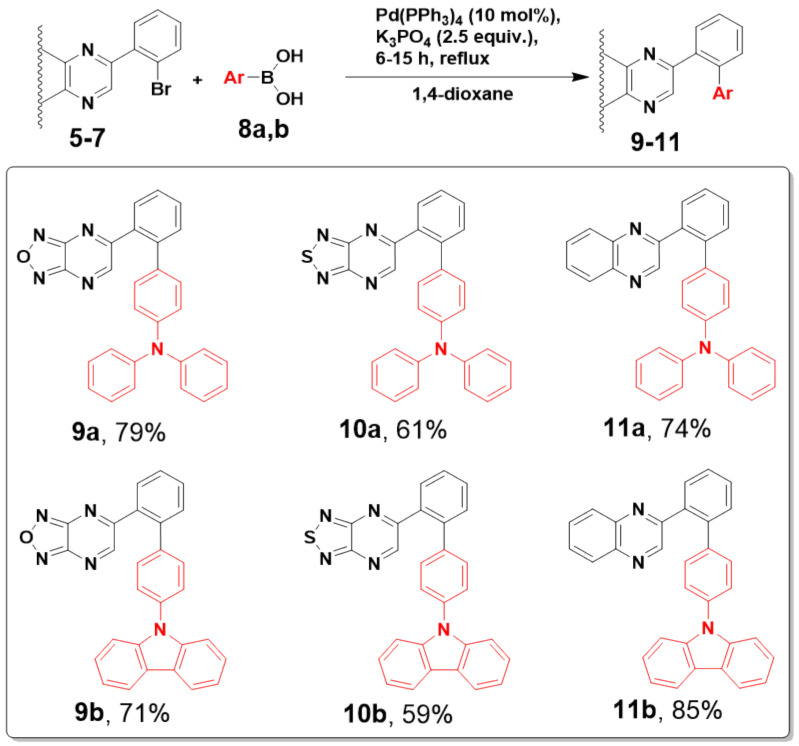
Synthesis and structure of V-shaped push–pull chromophores **9**–**11**.

The target V-shaped push–pull 5-(4′-(*N*,*N*-diphenylamino)-[1,1′-biphenyl]-2-yl)-[1,2,5]oxadiazolo[3,4-*b*]pyrazine (**9a**) and 5-(4′-(9*H*-carbazol-9-yl)-[1,1′-biphenyl]-2-yl)-[1,2,5]oxadiazolo[3,4-*b*]pyrazine (**9b**) were prepared through two-step procedure according an early developed synthetic approach starting from commercially available 2′-bromoacetophenone (**1**) (Scheme 1 and Scheme 2) [39].

In order to study the effect of the electron-withdrawing part on photophysical and electrochemical properties of the push–pull system, [1,2,5]thiadiazolo[3,4-*b*]pyrazine (**10a**,**b**) and quinoxaline (**11a**,**b**) structural analogues were synthesized. In this case, 1,2,5-thiadiazole-3,4-diamine (**3**) and *o*-phenylenediamine (**4**) were initially used to obtain corresponding 5-(2-bromophenyl)-[1,2,5]thiadiazolo[3,4-*b*]pyrazine (**6**) or 2-(2-bromophenyl)quinoxaline (**7**) with high yields (Scheme 1). All compounds were characterized by ^1^H/^13^C NMR and IR spectra as well as elemental analyses, and the results confirmed their structure.

In the second step, the desired chromophores **10a**,**b** and **11a**,**b** were obtained in good yields through the Suzuki–Miyaura cross-coupling reactions with the appropriate boronic acids **8a,b**, as shown in Scheme 2.

### 2.2. Electrochemical Properties

**Figure 3:**
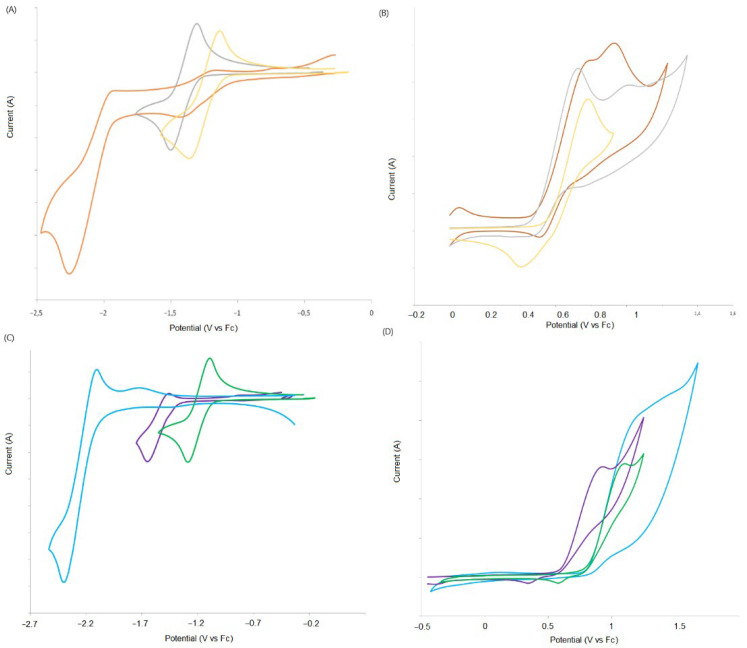
Cyclic voltamograms in DCM of compounds **9a** (yellow), **10a** (grey), and **11a** (red) in negative (**A**) and positive (**B**) direction and of **9b** (green), **10b** (purple), and **11b** (blue) in negative (**C**) and positive (**D**) direction.

Electrochemical behavior of compounds **9**–**11** were studied by cyclic voltammetry in dicholromethane (DCM) containing Bu_4_NPF_6_ electrolyte at a scan rate of 0.1 V/s. The working electrode was a glassy carbon disk. A Pt wire was used as the counter electrode, and an Ag wire as a reference electrode. Ferrocene was used as an internal reference for potential measurements. The first oxidation/reduction peak potentials and their differences are listed in Table 1. Representative CV diagrams of compounds are shown in Figure 3.

Compounds **9b**, **10b**, and **11b** bearing a 9*H*-carbazol-9-yl fragment as an electron-donating group exhibit a first irreversible oxidation process at, respectively, 1.08 V, 0.87 V, and 1.10 V vs. Fc/Fc^+^. The measured maximum current intensity is high in comparison with the values obtained for reduction processes, indicating a possible polymerization reaction on the electrode. As a consequence, an irreversible reduction broad peak is detected between −0.67 and −0.84 V on the reverse scan only after the oxidation of compounds (Figure S1). Upon initial scanning in the negative direction, a reversible reduction is observed at −0.95 and −1.10 V, respectively, for **9b** and **10b**, and an irreversible reduction is observed at *E*_pc_ = −2.36 V for **11b**. When a donor is a diphenylamino group (compounds **9a**, **10a**, and **11a**), the first irreversible oxidation is shifted towards lower potentials as compared to the respective carbazole analogues **9b**, **10b**, and **11b**. This confirms the superior electron-donating strength of the diphenylamino moiety. For these compounds, the first oxidation is followed by a second one at a higher potential (Figure S2). The modification of the donor has a very weak influence on the reduction of the diazine (see Table 1 and Figure 3). These trends indicate a weak ground-state interaction between the donor and acceptor.

When comparing the reduction process of the electron-withdrawing fragments, the reduction potential increase in the following order **11** < **10** < **9**, indicating that the [1,2,5]oxadiazolo[3,4-*b*]pyrazine electron-withdrawing fragment is the strongest one. As a result, compound **9a,** bearing the strongest electron-donating and electron-withdrawing parts, showed the lowest electronic gap (Δ*E*) and is expected to exhibit the most red-shifted absorption bands across the whole series. Compared to their 1,4-phenylene analogues previously described [39], compounds **9a** and **9b** exhibited a larger electronic gap (0.65 V and 0.48 V) due to more difficult both oxidation and reduction processes, which indicates a significantly lower ICT.

### 2.3. Photophysical Properties

**Figure 4:**
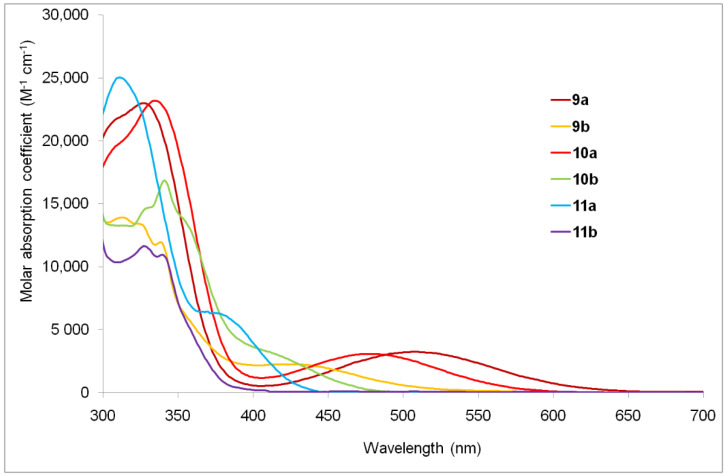
Absorption spectra or compounds **9**–**11** in DCM.

The UV-Vis and photoluminescence (PL) spectra of compounds **9**–**11** were measured in DCM solution at room temperature and the data are reported in Table 2. The UV/Vis spectra are provided in Figure 4. Regarding the lowest energetic absorption band, as expected according to electrochemical studies, the diphenylamino derivatives exhibit red-shifted absorption compared to their carbazole analogues. Similarly, as far as the electron-withdrawing part is concerned, the absorption maxima increase in the following order: **11** < **10** < **9** in both the diphenylamino and carbazole series. Compounds **9** and **10** are not luminescent in DCM solution, whereas the quinoxaline derivatives **11a** and **11b** exhibited green and yellow emissions, respectively, with low quantum yield. The particularly large Stokes shifts (>12,000 cm^−1^ for **11b**) could indicate the presence of a TICT excited state.

**Figure 5:**
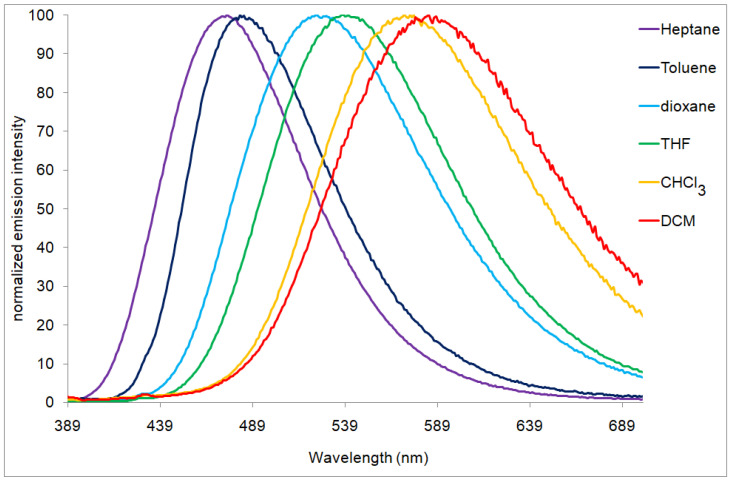
Normalized emission spectra of compound **11a** in a series of aprotic solvents (*c* = 1 × 10^−5^ M).

**Figure 6:**
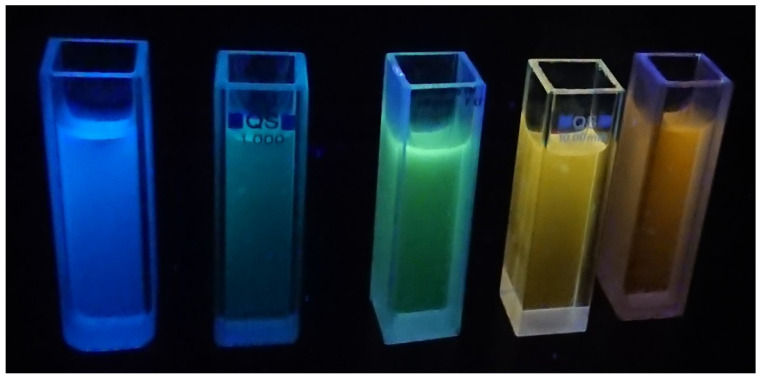
Fluorescence color change experienced by **11a** in various solvents (from left to right: *n*-heptane, toluene, 1,4-dioxane, CHCl_3_, DCM). The picture was taken in the dark upon irradiation with a handheld UV lamp (λ_em_ = 366 nm).

In an effort to gain further insight into the photophysical process within push–pull derivatives **11**, their emission spectra were registered in a series of aprotic solvents of increasing polarity. The positions of the corresponding emission maxima are reported in Table 3, and the normalized emission spectra registered for **11a**, as well as a picture of the solution in the various solvents, can be seen in Figure 5 and Figure 6 (the same data are presented for compound **11b** in Figures S3 and S4). Whereas the position of the absorption maximum is not significantly affected by the polarity of the solvent, the position of the emission maxima is bathochromically shifted when the polarity of the solvent, which is estimated according to the Reichardt polarity scale [44], is increased. This pronounced positive emission solvatochromism is characteristic of ICT and is well documented for push–pull materials [45,46,47,48], in particular in the case of biphenylene linkers between **A** and **D** parts [49]. Large emission solvatochromism and low emission quantum yield in a polar solvent are characteristics of TICT [50,51].

**Figure 7:**
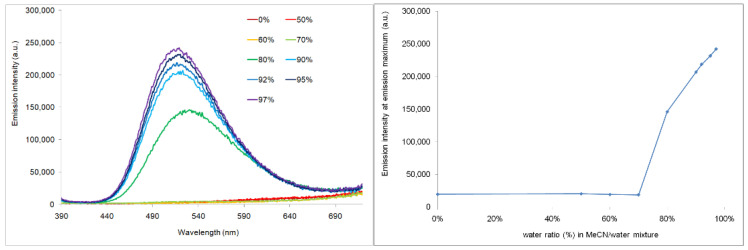
Left: Emission spectra of compound **11a** in MeCN/water mixture (*c* = 1.5 × 10^−5^ M, λ_exc_ = 374 nm), right: emission intensity of **11a** at emission maximum vs water ration in MeCN/water mixture.

**Figure 8:**
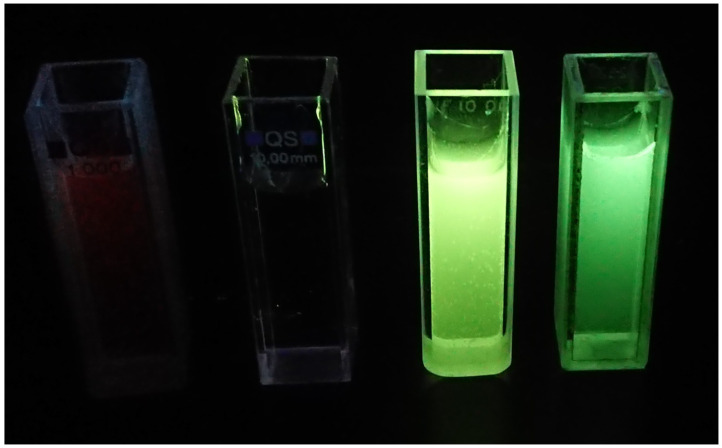
Fluorescence color of compound **11a** in MeCN/water mixture (from left to right: 0% 50%, 80% 97% of water) The picture was taken in the dark upon irradiation with a handheld UV lamp (λ_em_ = 366 nm).

Aggregation-induced emission (AIE), a concept proposed by Tang and coworkers in 2001 [52,53], induces intensive emission in the solid-state of non- (or slightly) emissive chromophores in solution. A restriction of intramolecular motion is one of the mechanisms of AIE [53]. In order to study the potential AIE properties of compounds **11**, their emission spectra were recorded in a mixture of MeCN and water of a different ratio (Figure 7 and Figure S5). The pictures of the solutions under UV irradiation are presented in Figure 8 and Figure S6. For **11a**, no emission is observed in pure MeCN, while a dramatic increase in the emission intensity is observed when the water fraction is higher than 80%, inducing a new band centered at 519 nm. The highest intensity is observed for the water fraction of 97%. Compound **11b** is slightly emissive in pure MeCN with a yellow emission centered at 570 nm. When the ratio of water is increased, an extinction of the emission is initially observed (a water fraction of 50/60%), followed by the appearance of a new, blue-shifted emission band (*λ*_max_ = 472 nm) with the maximum intensity of 80% of water. For higher water fractions, the emission is slightly blue-shifted and lowered in intensity.

**Figure 9:**
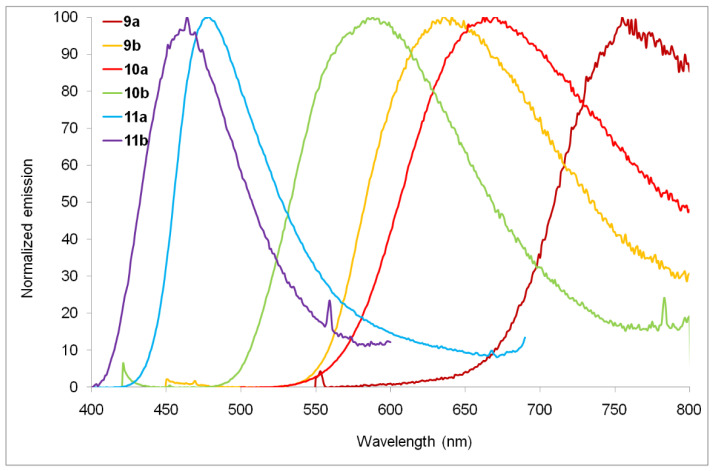
Normalized emission spectra of compounds **9**–**11** in KBr matrix (2 wt%).

**Figure 10:**
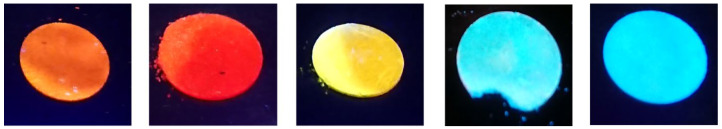
Emission color of KBr pellets doped with 2% of chromophores (from left to right: **9b**, **10a**, **10b**, **11a**, **11b**) The picture was taken in the dark upon irradiation with a handheld UV lamp (λ_exc_ = 366 nm).

The compounds **9**–**11** are luminescent solids as measured in the KBr matrix. The emission maxima are listed in Table 2. The spectra and pictures of selected KBr pellets are presented in Figure 9 and Figure 10. The KBr pellets exhibited intense emission from the blue for compound **11b** to near-infrared for compound **9a**. The emission is red-shifted in the same order as the emission bands recorded in solution.

### 2.4. NLO Properties

The second-order NLO properties of compounds **9**–**11** have been investigated in chloroform using the electric-field induced second harmonic generation (EFISH) method at a non-resonant incident wavelength of 1907 nm. The EFISH method provides an estimation of the NLO response as the scalar product between the permanent dipolar moment of the molecule $\vec{µ}$ and the vector component of, *β* described as *β_//_* [54,55,56]. The data are reported in Table 4. The NLO responses of compounds **9**–**11** are particularly low and we are close to the limit of detection of the system. It is therefore not reasonable to compare the values between them. For the 1,4-phenylene analogue of compounds **9a**, a much higher *µβ* value was measured (700 × 10^−48^ esu) [39]. This indicates that the 1,2-phenylene arrangement drastically reduces the NLO response due to the limited/diminished ICT.

### 2.5. Theoretical Calculation

Molecular structures and electronic properties of V-shaped push–pull chromophores **9**–**11** were theoretically investigated using the Gaussian 16 software package [57]. First, the geometries were optimized using the DFT B3LYP/6-311+G(2df,p) method in CHCl_3_. Using the same level of theory, the energies of the frontier molecular orbitals (HOMO/LUMO), ground-state dipole moments (*μ*), and first hyperpolarizabilities (*β*) were subsequently calculated. All the calculated data are gathered in Table 5; see the ESI for further information.

**Figure 11:**
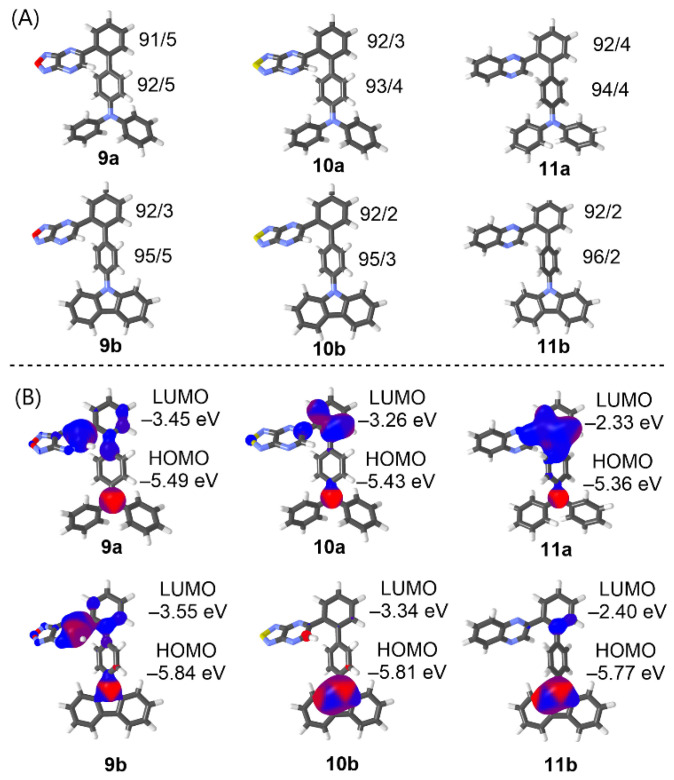
DFT-optimized geometries including Bird index/quinoid character of the particular 1,4-phenylene moieties (**A**) and HOMO (red) and LUMO (blue) localizations (**B**) in chromophores **9**–**11**.

The optimized geometries of **9**–**11** are shown in Figure 11A. As can be seen, the central biphenylene π-linker, interconnecting the *N*,*N*-diphenylamino, and pyrazine-derived moieties, adopts twisted geometry with the central torsion angle between 20 and 60°. Carbazole derivatives **b** showed generally larger torsion angles. The heterocyclic moiety at position 2 is always turned out by about 45°, independently of the particular derivative. The bond length alternation of both 1,4-phenylene moieties of the π-linker was investigated, revealing the Bird index *I*_6_ and the quinoid character (*δr*) within the range of 91–96/2–5% [58,59]. For unsubstituted benzene, the *I*_6_ and δ*r* are equal to 100 and 0, respectively. The calculated values imply that both 1,4-phenylene moieties in **9**–**11** are slightly polarized. Alternation of the heterocyclic acceptor significantly affected the ground state dipole moment of V-shaped chromophores. The largest ones were calculated for furazanopyrazine derivatives **9**, while replacement of the fused terminal oxa/thiadiazolo fragment by benzene diminished the dipole moment significantly as seen for **11**.

The calculated energies of the frontier molecular orbitals (*E*_HOMO/LUMO_) and their differences *E* are summarized in Table 5. The latter quantity correlates tightly with the electrochemical gaps (Figure S7). The oxadiazolopyrazine acceptor moiety in **9a**–**b** imparts the strongest ICT with the lowest calculated HOMO–LUMO gaps as compared to chromophores **10** and **11**. When comparing the triads of chromophores **a** and **b**, *N*,*N*-diphenylamino-substituted derivatives **a** possess lower HOMO–LUMO gaps than the corresponding carbazole derivatives **b**. Figure 11B shows HOMO and LUMO localizations in the particular derivatives; an obvious charge-separation is seen for **9a** and **9b**. For *N*,*N*-diphenylamino-substituted derivatives **10a** and **11a** are the situation similar, which is in contrast to carbazoles **10b** and **11b**. In these derivatives, both HOMO and LUMO are predominantly cumulated over the carbazole’s nitrogen atom, which results in their diminished ICT character and larger Δ*E* values.

Fundamental optical properties of **9**–**11** were investigated by TD-DFT CAM-B3LYP/6-311+G(2df,p) (nstates = 8) method. The calculated electronic absorption spectra along with the experimental ones are visualized in Figure S8. Both spectra feature the same shape and number of peaks and mostly differ in the position of the longest-wavelength absorption maxima. The *λ*_max_ values listed in Table 5 showed a very tight correlation with the experimental ones (Table 1)—see Figure S9. According to the aforementioned discussion on the HOMO–LUMO gap, oxadiazolopyrazine chromophores **9a**–**b** possess the most bathochromically shifted absorption maxima. Hence, both O→S replacement and fusing of benzene rings as in **10** and **11** shift the absorption maxima hypsochromically. An inspection of the transition forming the particular bands revealed that the longest wavelength absorption bands of chromophores **9a**–**b**, **10a**, and **11a** are generated by the HOMO→LUMO transition and these can be designed as CT bands. The corresponding high-energy bands (blue shifted) involve also the HOMO–1→LUMO and the HOMO→LUMO+1 transitions. On the contrary, the absorption of chromophores **10b** and **11b** is dominated by the HOMO–2→LUMO transition, with a weak contribution of the HOMO→LUMO transition seen for **10b**. These observations agree with their largest HOMO–LUMO gaps and the most hypsochromically shifted spectra.

The first order hyperpolarizability *β*(–2ω, ω, ω) calculated at 1907 nm is listed in Table 5. As can be seen, the largest polarizabilities were calculated for chromophores **9a** and **10a,** similarly to the EFISH experiment. Oxadiazolopyrazine, or eventually thiadiazolopyrazine, in combination with an *N*,*N*-diphenylamino substituent, is the most useful electron acceptor/donor pair in V-shaped NLOphores **9**–**11**.

## 3. Experimental Methods

### 3.1. General Information

All reagents and solvents were obtained from commercial sources and dried by using standard procedures before use. Starting materials **5** and **7** were prepared according to the earlier reported procedure [60]. The ^1^H and ^13^C NMR spectra were recorded on a Bruker AVANCE-600 instruments using Me_4_Si as an internal standard. Elemental analysis was carried on a Eurovector EA 3000 automated analyzer. Melting points were determined on Boetius combined heating stages and were not corrected. The chromatographic purification of compounds was achieved with silica gel Alfa Aesar 0.040–0.063 mm (230–400 mesh), eluting with CH_2_Cl_2_/hexane (1:2, *v*/*v*). The progress of reactions and the purity of compounds were checked by TLC on Sorbfil plates (Russia), in which the spots were visualized with UV light (λ 254 or 365 nm). IR spectra of samples (solid powders) were recorded on a Spectrum One Fourier transform IR spectrometer (Perkin Elmer, Waltham, MA, USA) equipped with a diffuse reflectance attachment (DRA) in the frequency range 4000 ÷ 400 cm^−1^. Spectrum processing and band intensity determination were carried out using the special software supplied with the spectrometer.

### 3.2. Electrochemical Characterization

The electrochemical studies of the compounds were performed with a home-designed 3-electrodes cell (WE: glassy carbon disk, RE: Ag wire, Ce: Pt). Ferrocene was added at the end of each experiment to determine redox potential values.

### 3.3. Photophysical Characterization

The absorption spectra of the samples were detected with a JASCO V-650 instrument, whereas the emission spectra were detected by a Horiba Fluoromax spectrophotometer. UV/Vis and fluorescence spectra were recorded by using standard 1 cm quartz cells. Compounds were excited at their absorption maxima in solution (band of lowest energy) to record the emission spectra. The *Φ_F_* values were calculated by using a well-known procedure with 9,10-diphenylethynylanthracene in cyclohexane as a standard (*Φ_F_* = 1.00) [61]. Stokes shifts were calculated by considering the lowest energy absorption band. Experimental details on EFISH measurements are described elsewhere [62].

**Synthesis of 5-(2-bromophenyl)-[1,2,5]thiadiazolo[3,4-*b*]pyrazine (6)**. A mixture of 2′-bromoacetophenone **1** (1.994 g, 10 mmol) and selenium dioxide (1.1 g, 10 mmol) in a solution of 1,4-dioxane (15 mL) and water (1 mL) was refluxed for 12 h. Selenium was filtered off and washed with 1,4-dioxane (5 mL). The solvent was evaporated at reduced pressure. The residue was dissolved in a mixture of ethanol (5 mL) and acetic acid (5 mL), 1,2,5-thiadiazole-3,4-diamine (**3**) (1.16 g, 10 mmol) was added, and the resulting mixture was refluxed for 1 h and cooled to room temperature. A precipitate formed was filtered, washed with ethanol, and dried in air. Compound **6** was obtained as a pale-yellow solid. Yield 2.2 g (75%), mp 169–170 °C. The ^1^H NMR (600 MHz, DMSO-*d*_6_) δ 9.45 (s, 1H), 7.90 (dd, *J* = 8.1, 1.1 Hz, 1H), 7.79 (dd, *J* = 7.6, 1.7 Hz, 1H), 7.66 (td, *J* = 7.5, 1.1 Hz, 1H), 7.57 (td, *J* = 7.7, 1.7 Hz, 1H). The ^13^C NMR (151 MHz, DMSO-*d*_6_) δ 158.2, 154.0, 153.4, 151.7, 137.3, 133.7, 132.8, 132.7, 128.9, 121.7. Calcd. For C_10_H_5_BrN_4_S (293.14): C, 40.97; H, 1.72; N, 19.11. Found: C, 41.02; H, 1.77; N, 19.16.

**General procedure for the synthesis of 5-(4′-(heteroaryl)-[1,1′-biphenyl]-2-yl)-[1,2,5]oxadiazolo[3,4-*b*]pyrazine (9), 5-(4′-(heteroaryl)-[1,1′-biphenyl]-2-yl)-[1,2,5]thiadiazolo[3,4-*b*]pyrazine (10), and 2-(4′-(heteroaryl)-[1,1′-biphenyl]-2-yl)- quinoxaline (11).** A mixture of 5-(2-bromophenyl)-[1,2,5]oxadiazolo[3,4-*b*]pyrazine (5) (277 mg, 1.0 mmol), 5-(2-bromophenyl)-[1,2,5]thiadiazolo[3,4-*b*]pyrazine (6) (293 mg, 1.0 mmol) or 2-(2-bromophenyl)quinoxaline (7) (285 mg, 1.0 mmol)], corresponding arylboronic acid 8a or 8b (1.2 mmol), Pd(PPh_3_)_4_ (115 mg, 10 mol %) and K_3_PO_3_ (530 mg, 2.5 mmol) were dissolved in 1,4-dioxane (15 mL). The reaction mixture was degassed and refluxed for 15 h under an argon atmosphere. After completion of the reaction (as monitored by TLC), the reaction mixture was cooled, filtered, and dissolved in a mixture of EtOAc and water (1:1, 50 mL). The organic layer was separated, the aqueous layer was extracted with EtOAc (2 × 25 mL), the combined organic extracts were dried with MgSO_4_ and the solvents were evaporated. Purification by silica gel column chromatography with CH_2_Cl_2_/hexane (1:2, *v*/*v*) as an eluent afforded the title compounds 9–11.

**2′-([1,2,5]Oxadiazolo[3,4-*b*]pyrazin-5-yl)-*N,N*-diphenyl-[1,1′-biphenyl]-4-amine (9a)**. Yield 348 mg (79%), dark violet solid, mp 160–161 °C. The ^1^H NMR (600 MHz, DMSO-*d*_6_) δ 8.31 (s, 1H), 7.91 (d, *J* = 7.7 Hz, 1H), 7.76 (t, *J* = 7.5 Hz, 1H), 7.69–7.63 (m, 2H), 7.32 (t, *J* = 7.7 Hz, 4H), 7.17 (d, *J* = 8.2 Hz, 2H), 7.10–7.04 (m, 6H), 6.91 (d, *J* = 8.2 Hz, 2H). The ^13^C NMR (151 MHz, DMSO-*d*_6_) δ 164.5, 157.3, 152.8, 151.3, 148.0, 147.1, 141.4, 134.4, 132.7, 132.5, 131.8, 131.6, 130.5, 130.1, 128.5, 125.1, 124.2, 122.8. Calcd. for C_28_H_19_N_5_O (441.49): C, 76.17; H, 4.34; N, 15.86. Found: C, 76.24; H, 4.46; N, 15.70. ν (DRA, cm^−1^) 3061 (w, C–H_Ar_), 3036 (w, C–H_Ar_), 1588 (s, C–C_A_/C–N_Ar_), 1487 (s, C–C_Ar_/C–N_Ar_), 1443 (s, C–C_Ar_/C–N_Ar_), 753 (s, C–H_Ar_), 695 (s, C–H_Ar_).

**5-(4′-(9*H*-Carbazol-9-yl)-[1,1′-biphenyl]-2-yl)-[1,2,5]oxadiazolo[3,4-*b*]pyrazine (9b)**. Yield 311 mg (71%), red-orange solid, mp 226–227 °C. The ^1^H NMR (600 MHz, DMSO-*d*_6_) δ 8.50 (s, 1H), 8.26 (dt, *J* = 7.8, 0.9 Hz, 2H), 8.00 (dd, *J* = 7.7, 1.2 Hz, 1H), 7.88–7.82 (m, 2H), 7.75 (ddd, *J* = 7.7, 6.9, 1.8 Hz, 1H), 7.65–7.62 (m, 2H), 7.60–7.56 (m, 2H), 7.46 (ddd, *J* = 8.3, 7.1, 1.2 Hz, 2H), 7.38 (dt, *J* = 8.2, 0.9 Hz, 2H), 7.31 (ddd, *J* = 7.9, 7.1, 1.0 Hz, 2H). The ^13^C NMR (151 MHz, DMSO-*d*_6_) δ 164.2, 157.3, 152.9, 151.4, 141.0, 140.4, 138.6, 137.3, 134.8, 132.7, 132.3, 132.1, 131.0, 129.2, 127.6, 126.8, 123.4, 121.1, 120.8, 110.0. Calcd. for C_28_H_17_N_5_O (439.48): C, 76.52; H, 3.90; N, 15.94. Found: C, 76.47; H, 3.78; N, 15.82. ν (DRA, cm^−1^) 3060 (w, C–H_Ar_), 1600 (s, C–C_Ar_/C–N_Ar_), 1450 (s, C–C_Ar_/ C–N_Ar_), 749 (s, C–H_Ar_), 724 (s, C–H_Ar_).

**2′-([1,2,5]Thiadiazolo[3,4-*b*]pyrazin-5-yl)-*N,N*-diphenyl-[1,1′-biphenyl]-4-amine (10a)**. Yield 278 mg (61%), bright orange solid, mp 189–190 °C. The ^1^H NMR (600 MHz, DMSO-*d*_6_) δ 8.48 (s, 1H), 7.89 (dd, *J* = 7.7, 1.3 Hz, 1H), 7.71 (td, *J* = 7.5, 1.4 Hz, 1H), 7.63 (ddd, *J* = 14.9, 7.6, 1.3 Hz, 2H), 7.34–7.29 (m, 4H), 7.13–7.09 (m, 2H), 7.08–7.04 (m, 2H), 7.04–7.00 (m, 4H), 6.89–6.85 (m, 2H). The ^13^C NMR (151 MHz, DMSO-*d*_6_) δ 159.9, 154.5, 152.8, 152.1, 147.5, 147.2, 141.2, 135.1, 133.5 131.7, 131.5, 131.5, 130.6, 130.1, 128.4, 124.9, 124.0, 123.0. Calcd. for C_28_H_19_N_5_S (457.56): C, 73.50; H, 4.19; N, 15.31. Found: C, 73.23; H, 4.24; N, 15.21. ν (DRA, cm^−1^) 3085 (w, C–H_Ar_), 3064 (w, C–H_Ar_), 3025 (w, C–H_Ar_), 1594 (s, C–C_A_/C–N_Ar_), 1483 (s, C–C_Ar_/C–N_Ar_), 754 (s, C–H_Ar_), 693 (s, C–H_Ar_).

**5-(4′-(9*H*-Carbazol-9-yl)-[1,1′-biphenyl]-2-yl)-[1,2,5]thiadiazolo[3,4-*b*]pyrazine (10b)**. Yield 268 mg (59%), yellow solid, mp 250–251 °C. The ^1^H NMR (600 MHz, DMSO-*d*_6_) δ 8.65 (s, 1H), 8.24 (d, *J* = 7.8 Hz, 2H), 7.98 (d, *J* = 7.6 Hz, 1H), 7.80 (q, *J* = 5.1, 4.2 Hz, 2H), 7.73 (td, *J* = 6.8, 5.7, 2.9 Hz, 1H), 7.61–7.48 (m, 4H), 7.44 (t, *J* = 7.6 Hz, 2H), 7.36–7.25 (m, 4H). The ^13^C NMR (151 MHz, DMSO-*d*_6_) δ 159.5, 154.5, 152.9, 152.0, 140.8, 140.4, 139.2, 136.8, 135.4, 132.1, 132.0, 131.7, 131.0, 129.1, 127.4, 126.7, 123.3, 121.1, 120.7, 110.0. Calcd. for C_28_H_17_N_5_S (455.54): C, 73.83; H, 3.76; N, 15.37. Found: C, 73.70; H, 3.75; N, 15.24. ν (DRA, cm^−1^) 3060 (w, C–H_Ar_), 3042 (w, C–H_Ar_), 1597 (s, C–C_Ar_/C–N_Ar_), 1452 (s, C–C_Ar_/ C–N_Ar_), 751 (s, C–H_Ar_), 725 (s, C–H_Ar_).

***N,N*-Diphenyl-2′-(quinoxalin-2-yl)-[1,1′-biphenyl]-4-amine (11a)**. Yield 332 mg (74%), pale yellow solid, mp 135–137 °C. The ^1^H NMR (600 MHz, DMSO-*d*_6_) δ 8.41 (s, 1H), 8.07 (ddd, *J* = 8.5, 7.1, 1.6 Hz, 2H), 7.89–7.82 (m, 3H), 7.63 (td, *J* = 7.1, 6.6, 1.5 Hz, 1H), 7.60–7.55 (m, 2H), 7.30–7.23 (m, 4H), 7.02 (dd, *J* = 7.9, 5.9 Hz, 4H), 6.99–6.95 (m, 4H), 6.86–6.80 (m, 2H). The ^13^C NMR (151 MHz, DMSO-*d*_6_) δ 154.9, 147.3, 147.0, 146.9, 142.1, 140.9, 140.4, 136.4, 134.6, 131.4, 131.2, 130.7, 130.5, 130.40, 130.38, 130.0, 129.5, 129.3, 128.3, 124.4, 123.7, 123.4. Calcd. for C_32_H_23_N_3_ (449.56): C, 85.50; H, 5.16; N, 9.35. Found: C, 85.49; H, 5.36; N, 9.29. ν (DRA, cm^−1^) 3057 (w, C–H_Ar_), 3034 (w, C–H_Ar_), 1585 (s, C–C_A_/C–N_Ar_), 1492 (s, C–C_Ar_/C–N_Ar_), 1481 (s, C–C_Ar_/C–N_Ar_), 752 (s, C–H_Ar_), 694 (s, C–H_Ar_).

**9-(2′-(Quinoxalin-2-yl)-[1,1′-biphenyl]-4-yl)-9*H*-carbazole (11b)**. Yield 379 mg (85%), pale yellow solid, mp 158–159 °C. The ^1^H NMR (600 MHz, DMSO-*d*_6_) δ 8.56 (s, 1H), 8.25–8.21 (m, 2H), 8.08 (td, *J* = 8.5, 1.7 Hz, 2H), 7.93 (dd, *J* = 7.4, 1.1 Hz, 1H), 7.90–7.84 (m, 2H), 7.75–7.71 (m, 2H), 7.70–7.66 (m, 1H), 7.52–7.49 (m, 2H), 7.41 (dd, *J* = 8.9, 7.3 Hz, 4H), 7.31–7.26 (m, 4H). The ^13^C NMR (151 MHz, DMSO-*d*_6_) δ 154.6, 146.9, 142.1, 140.6, 140.5, 140.5, 139.9, 136.7, 136.4, 131.8, 131.5, 130.9, 130.8, 130.6, 130.5, 129.6, 129.3, 128.9, 127.2, 126.7, 123.2, 121.0, 120.6, 109.9. Calcd. for C_32_H_21_N_3_ (447.54): C, 85.88; H, 4.73; N, 9.39. Found: C, 85.95; H, 4.85; N, 9.41. ν (DRA, cm^−1^) 3055 (w, C–H_Ar_), 3042 (w, C–H_Ar_), 1598 (s, C–C_Ar_/C–N_Ar_), 1450 (s, C–C_Ar_/ C–N_Ar_), 748 (s, C–H_Ar_), 724 (s, C–H_Ar_).

## 4. Conclusions

In summary, we have designed a series of push–pull chromophores bearing quinoxaline, [1,2,5]oxadiazolo[3,4-*b*]pyrazine, and [1,2,5]thiadiazolo[3,4-*b*]pyrazine as A part and 2,4′-biphenylene as a π-conjugated linker. The [1,2,5]oxadiazolo[3,4-*b*]pyrazine fragment appears as the strongest A part according to experimental electrochemical and photophysical results and theoretical calculation. With regards to 4,4′-biphenylene and 1,4-phenylene-2,5-thienylene known analogues, these compounds exhibit reduced ICT leading to a low NLO response. Nevertheless, all compounds exhibited intense emissions in the solid state ranging from cyan to red/near-infrared. Quinoxaline derivatives **11** exhibited TICT emission with intense solvatochromism, large Stokes shifts, and AIE.

## Figures and Tables

**Table 1:** Electrochemical data of compounds **9**–**11**.

Compounds	*E*_pa_ [V] ^a^	*E*_1/2_^red^ [V] ^a^	Δ*E* [V] ^b^	*E*_HOMO_ [eV] ^c^	*E*_LUMO_ [eV] ^c^	*λ*_max_ [nm] ^d^
**9a**	0.79	−1.24	2.04	−5.59	−3.56	608
**9b**	1.08	−1.20	2.28	−5.88	−3.60	544
**10a**	0.72	−1.50	2.22	−5.52	−3.30	569
**10b**	0.87	−1.55	2.42	−5.67	−3.25	513
**11a**	0.74	−2.44	3.17	−5.54	−2.36	391
**11b**	1.10	−2.36 ^e^	3.46	−5.90	−2.44	359

^a^ All potentials are given versus Fc/Fc^+^. ^b^ Δ*E* = *E*_pa_ – *E*_1/2_^red1^.^c^ *E*_HOMO/LUMO_ = –(*E*^ox1/red1^ + 4.8). ^d^ Calculated *λ*_max_ values (*λ* = 1241/Δ*E*). ^e^ Irreversible peaks *E*_p_.

**Table 2:** Photophysical data of compounds **9**–**11** in DCM solution and in KBr matrix.

Compounds	DCM Solution			KBr Matrix
	*λ*_abs_ [nm] (ε [mM^−1^ cm^−1^])	*λ*_em_ [nm] (*Φ* ^a^)	Stoke Shift (cm^−1^)	*λ*_em_ [nm]
**9a**	327 (23.0), 508 (3.3)	- ^a^	- ^a^	755
**9b**	324 (13.4), 422 (2.3)	- ^a^	- ^a^	636
**10a**	335 (23.2), 479 (3.1)	- ^a^	- ^a^	662
**10b**	340 (16.8), 411 (3.2)	- ^a^	- ^a^	588
**11a**	311 (25.0), 374 (6.3)	512 (0.03)	7206	478
**11b**	326 (11.6), 340 (10.9)	584 (0.01)	12,288	464

^a^ 9,10-diphenylethynylanthracene in cyclohexane as a standard (*Φ_F_* = 1.00).

**Table 3:** Emission solvatochromism of compounds **11**.

Compounds	*λ*_em_ [nm] ^a^					
	*n*-Heptane E_T_(30) = 30.9 ^a^	Toluene E_T_(30) = 33.9 ^a^	1,4-Dioxane E_T_(30) = 36.0 ^a^	THF E_T_(30) = 37.4 ^a^	CHCl_3_ E_T_(30) = 39.1 ^a^	DCM E_T_(30) = 40.7 ^a^
**11a**	456	487	521	534	573	584
**11b**	422	431	452	484	495	512

^a^ Reichardt polarity parameter (kcal mol^−1^).

**Table 4:** Results of EFISH measurements for compounds **9–11**.

	9a	9b	10a	10b	11a	11b
*µβ* (10^−48^ esu)	80	20	80	50	40	0

**Table 5:** DFT-calculated data of compounds **9**–**11**.

Compounds	*E*_HOMO_^DFT^ [eV]	*E*_LUMO_^DFT^ [eV]	Δ*E*^DFT^ [eV]	*μ* [D]	*λ*_max_^TD-DFT^ [nm/eV]	*β*(–2ω,ω,ω)^DFT^ [10^−30^ esu]
**9a**	–5.49	–3.45	2.04	8.11	447/2.77	504
**9b**	–5.84	–3.55	2.29	7.86	377/3.29	117
**10a**	–5.43	–3.26	2.17	4.24	420/2.95	311
**10b**	–5.81	–3.34	2.47	4.30	357/3.47	74
**11a**	–5.36	–2.33	3.03	0.87	335/3.70	53
**11b**	–5.77	–2.40	3.37	2.37	301/4.12	18

All data calculated at the DFT level by using the Gaussian^®^ 16 software package and DFT B3LYP/6-311+G(2df,p) method in CHCl_3_. The first hyperpolarizabilities *β*(−2**ω**,**ω**,**ω**) were calculated at 1907 nm. The electronic absorption spectra, the longest-wavelength absorption maxima and the corresponding electron transitions were calculated using TD-DFT (*n*_states_ = 8) B3LYP/6-311+G(2df,p).

## Data Availability

The data are available on request from the corresponding authors.

## Supplementary Materials

The following supporting information can be downloaded at: https://www.mdpi.com/article/10.3390/molecules27134250/s1, Figures S1 and S2: Oxidation study of compounds **9b**, **10b**, **11a**, and **11b**. Figure S3: Normalized emission spectra of compound **11b** in a series of aprotic solvents. Figure S4: Fluorescence color change experienced by **11b** in various solvents. Figure S5: Emission spectra of compound **11b** in MeCN/water mixture. Figure S6: Fluorescence color of compound **11b** in MeCN/water mixture. Figure S7: A correlation of the experimental (ELCH) and DFT-calculated HOMO–LUMO gaps of chromophores **9**–**11**. Figure S8: TD-DFT (nstates = 8) B3LYP/6-311+G(2d,f,p) calculated UV-Vis spectra of chromophores **9**–**11** in CHCl_3_ (red curves) along with the experimental spectra (black curves). Red vertical lines represent oscillator strengths (f). Figure S9: A correlation of the experimental (EXP) and DFT-calculated *λ*_max_ values of chromophores **9**–**11**. Figures S10–S29: ^1^H ^13^C NMR and IR spectra of compounds **6**, **9**–**11**.

## Sample Availability

Samples of the compounds **9**–**11** are available from the authors.
