# Supplementary material for: Push–Pull Derivatives Based on 2,4′-Biphenylene Linker with Quinoxaline, [1,2,5]Oxadiazolo[3,4-B]Pyrazine and [1,2,5]Thiadiazolo[3,4-B]Pyrazine Electron Withdrawing Parts
Source: Molecules. 2022 Jun 30;27(13):4250. doi: 10.3390/molecules27134250 (PMC9268487; doi:10.3390/molecules27134250)
Supplement: Supplementary file 1 [file molecules-27-04250-s001.zip › molecules-1790596-supplementary.pdf]

SUPPORTING INFORMATION

Push-pull derivatives based on 2,4′-biphenylene linker with quinoxaline, [1,2,5]oxadiazolo[3,4-*b*]pyrazine and [1,2,5]thiadiazolo[3,4-*b*]pyrazine electron withdrawing parts

Egor V. Verbitskiy,[a,b,*] Pascal le Poul,[c] Filip Bureš,[d,*] Sylvain Achelle,[c,*] Alberto Barsella[e], Yuriy A. Kvashnin[a], Gennady L. Rusinov[a,b], Valery N. Charushin[a,b]

[a]*I. Postovsky Institute of Organic Synthesis, Ural Branch of the Russian Academy of Sciences, S. Kovalevskaya Str., 22, Ekaterinburg, 620108, Russia*

[b]*Ural Federal University, Mira St. 19, Ekaterinburg, 620002, Russia*

[c]*Univ Rennes, CNRS, Institut des Sciences Chimiques de Rennes – UMR 6226, 35000 Rennes, France*

[d]*Institute of Organic Chemistry and Technology, Faculty of Chemical Technology, University of Pardubice Studenská 573, Pardubice, 53210, Czech Republic*

[e]*Département d'Optique ultrarapide et Nanophotonique, IPCMS, UMR CNRS 7504, Université de Strasbourg, 23 rue du Loess, BP 43, 67034 Strasbourg Cedex 2, France*

Figure S1. Oxidation study of compounds **9b** (*green*), **10b** (*orange*) and **11b** (*blue*). 3
Figure S2. Oxidation study of compound **11a.**..3
Figure S3: Normalized emission spectra of compound **11b** in a series of aprotic solvents (c ~ 1×10^{-5} M)..4
Figure S4: Fluorescence color change experienced by **11b** in various solvents (from left to right: n-heptane, toluene, 1,4-dioxane, $CHCl_3$, DCM) The picture was taken in the dark upon irradiation with a handheld UV lamp (λ_{em} = 366 nm) 4
Figure S5: Emission spectra of compound **11b** in MeCN/water mixture ($c = 1.5 \times 10^{-5}$ M, λ_{exc} = 340 nm)..5
Figure S6: Fluorescence color of compound **11b** in MeCN/water mixture (from left to right: 0% 50%, 80% 97% of water) The picture was taken in the dark upon irradiation with a handheld UV lamp (λ_{em} = 366 nm). 5
Figure S7: A correlation of the experimental (ELCH) and DFT-calculated HOMO–LUMO gaps of chromophores **9**–**11**. 6
Figure S8: TD-DFT (*n*states = 8) B3LYP/6-311+G(2d,f,p) calculated UV-Vis spectra of chromophores **1a**–**9a** in $CHCl_3$ (red curves) along with the experimental spectra (black curves). Red vertical lines represent oscillator strengths (*f*). 7

Figure S9: A correlation of the experimental (EXP) and DFT-calculated λ_{max} values of chromophores **9**–**11**. 7
Figure S10. ^{1}H NMR (600 MHz, DMSO-d_6) spectrum of **6**. 8
Figure S11. ^{13}C NMR (151 MHz, DMSO-d_6) spectrum of **6**. 8
Figure S12. ^{1}H NMR (600 MHz, DMSO-d_6) spectrum of **9a**. 9
Figure S13. ^{13}C NMR (151 MHz, DMSO-d_6) spectrum of **9a**. 9
Figure S14. ^{1}H NMR (600 MHz, DMSO-d_6) spectrum of **9b**. 10
Figure S15. ^{13}C NMR (151 MHz, DMSO-d_6) spectrum of **9b**. 10
Figure S16. ^{1}H NMR (600 MHz, DMSO-d_6) spectrum of **10a**. 111
Figure S17. ^{13}C NMR (151 MHz, DMSO-d_6) spectrum of **10a**. 111
Figure S18. ^{1}H NMR (600 MHz, DMSO-d_6) spectrum of **10b**. 122
Figure S19. ^{13}C NMR (151 MHz, DMSO-d_6) spectrum of **10b**. 12
Figure S20. ^{1}H NMR (600 MHz, DMSO-d_6) spectrum of **11a**. 13
Figure S21. ^{13}C NMR (151 MHz, DMSO-d_6) spectrum of **11a**. 14
Figure S22. ^{1}H NMR (600 MHz, DMSO-d_6) spectrum of **11b**. 14
Figure S23. ^{13}C NMR (151 MHz, DMSO-d_6) spectrum of **11b**. 15
Figure S24. IR spectrum of **9a**. 15
Figure S25. IR spectrum of **9b**. 16
Figure S26. IR spectrum of **10a**. 16
Figure S27. IR spectrum of **10b**. 17
Figure S28. IR spectrum of **11a**. 17
Figure S29. IR spectrum of **11b**. 18
TD-DFT data of chromophore **9a**....................18
TD-DFT data of chromophore **9b**....................19
TD-DFT data of chromophore **10a**....................20
TD-DFT data of chromophore **10b**....................21
TD-DFT data of chromophore **11a**....................22
TD-DFT data of chromophore **11b**....................23

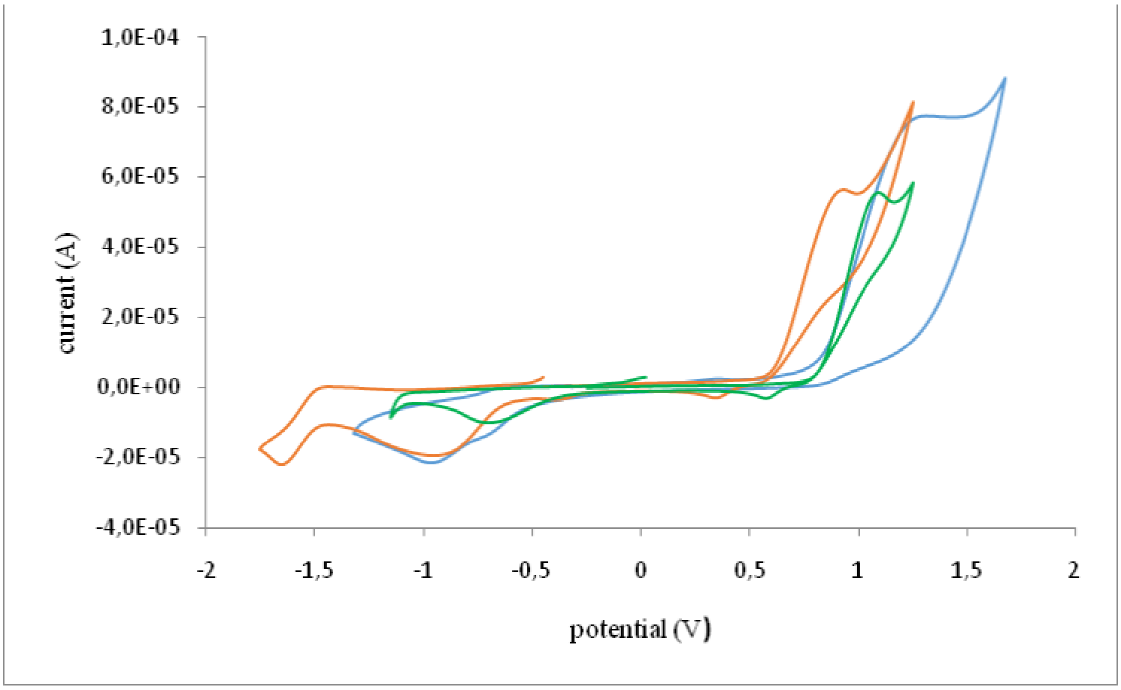

Figure S1. Oxidation study of compounds **9b** (*green*), **10b** (*orange*) and **11b** (*blue*).

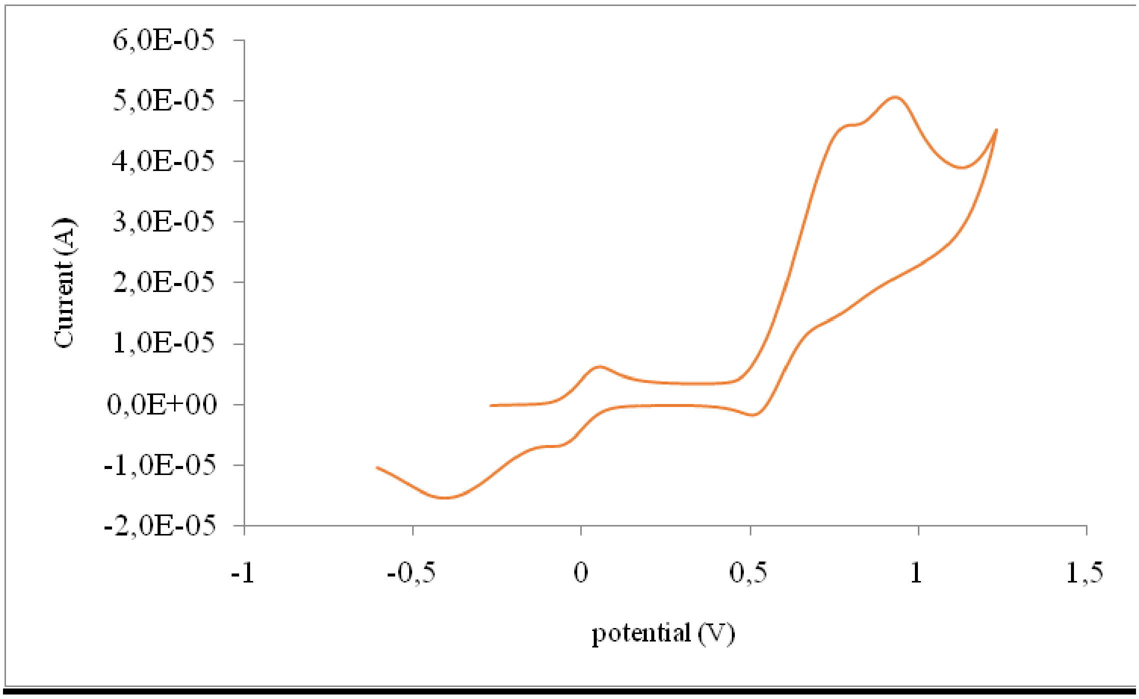

Figure S2. Oxidation study of compound **11a.**

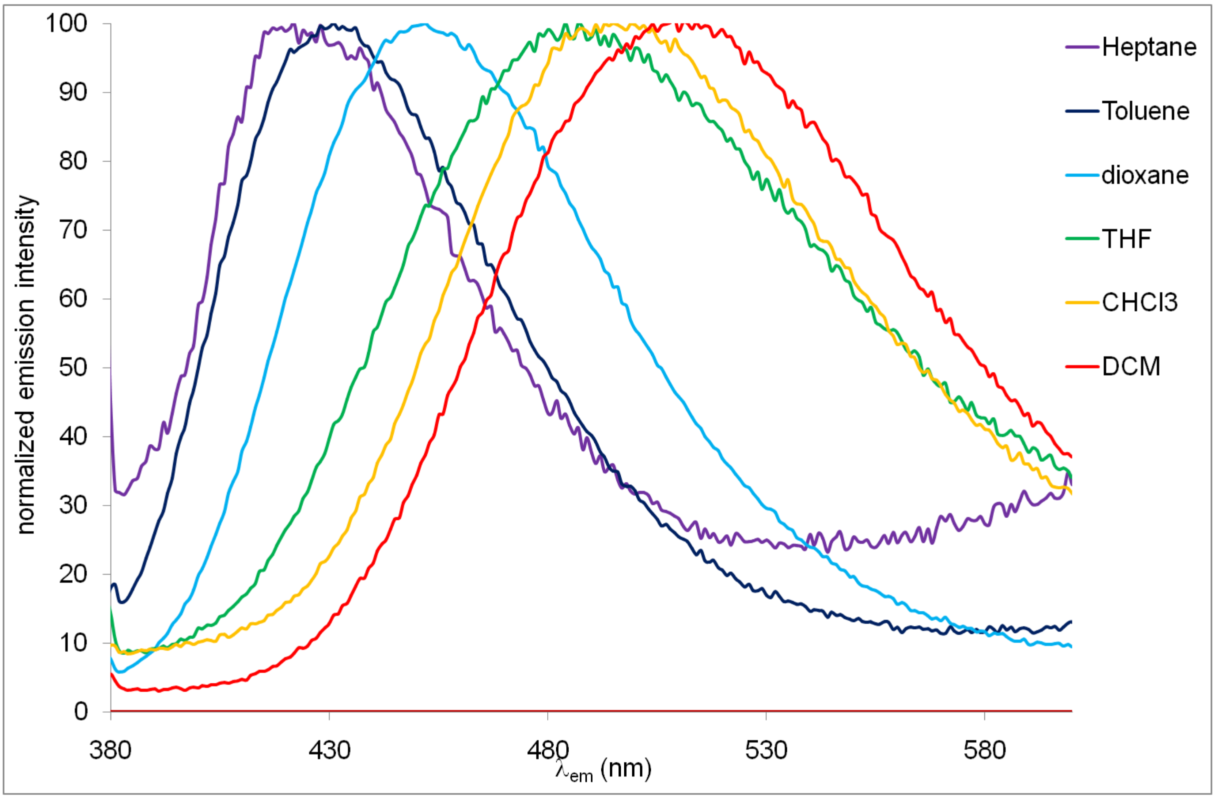

Figure S3: Normalized emission spectra of compound **11b** in a series of aprotic solvents (c ~ 1×10^{-5} M).

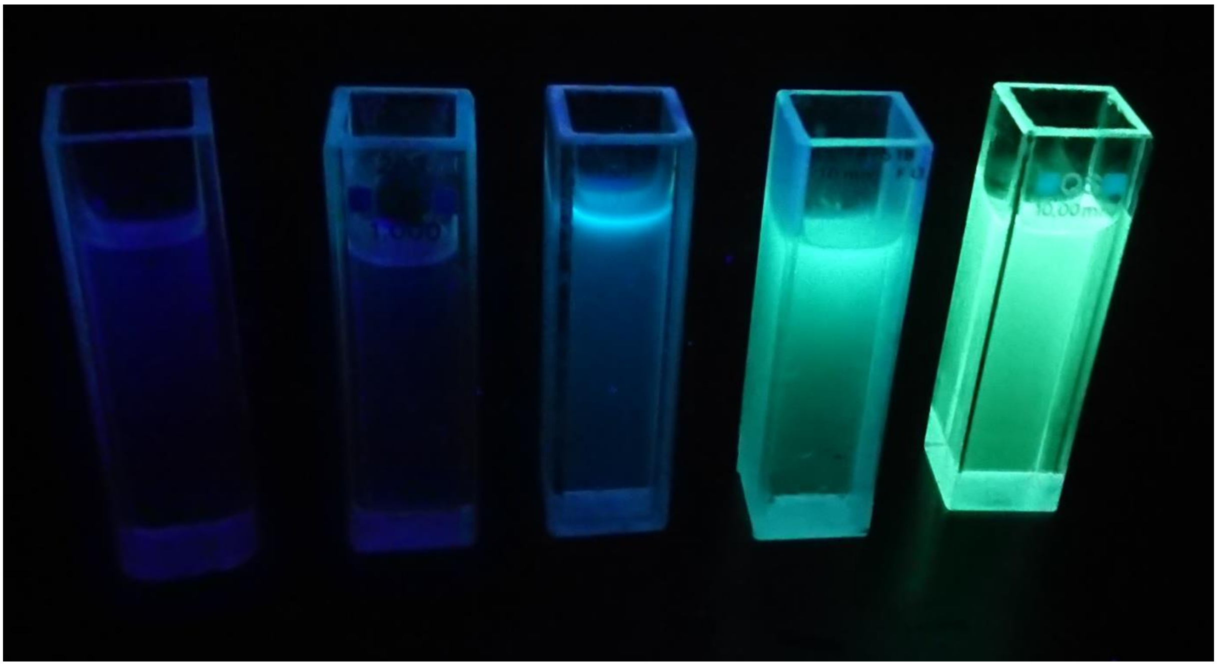

Figure S4: Fluorescence color change experienced by **11b** in various solvents (from left to right: n-heptane, toluene, 1,4-dioxane, $CHCl_3$, DCM) The picture was taken in the dark upon irradiation with a handheld UV lamp (λ_{em} = 366 nm)

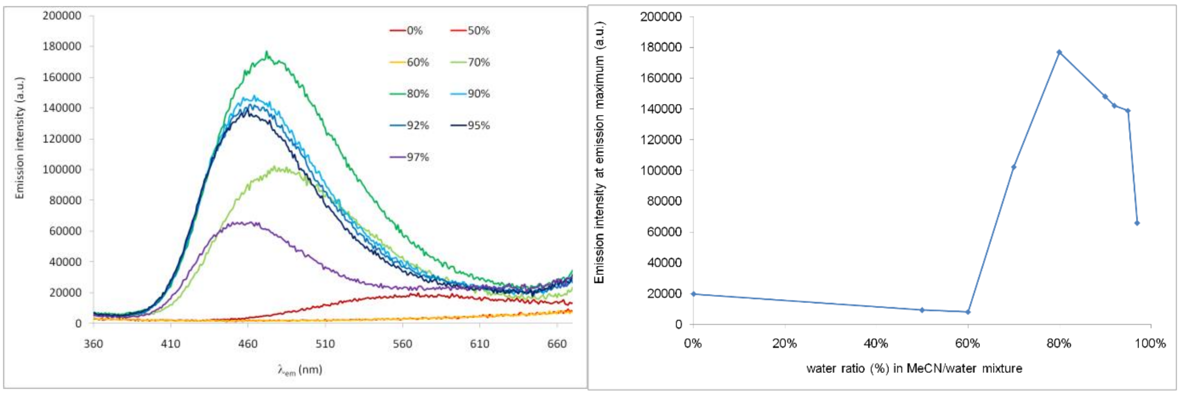

Figure S5: Emission spectra of compound **11b** in MeCN/water mixture (c = 1.5 × 10^{-5} M, λ_{exc} = 340 nm).

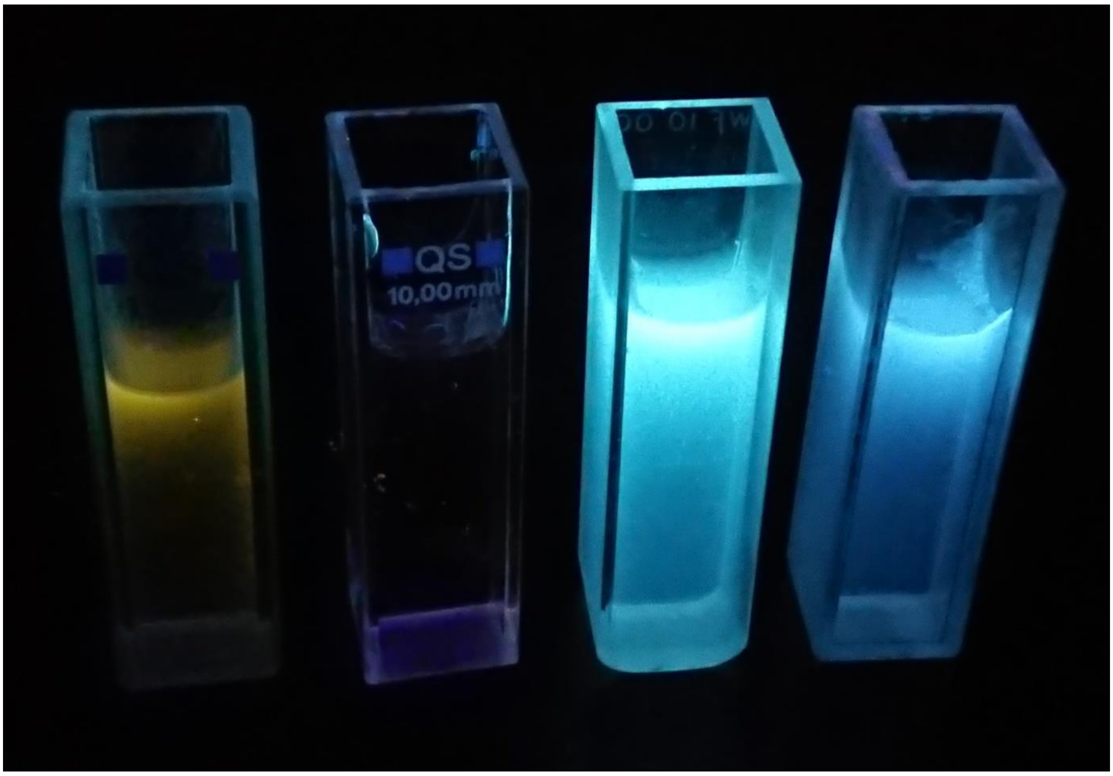

Figure S6: Fluorescence color of compound **11b** in MeCN/water mixture (from left to right: 0% 50%, 80% 97% of water) The picture was taken in the dark upon irradiation with a handheld UV lamp (λ_{em} = 366 nm).

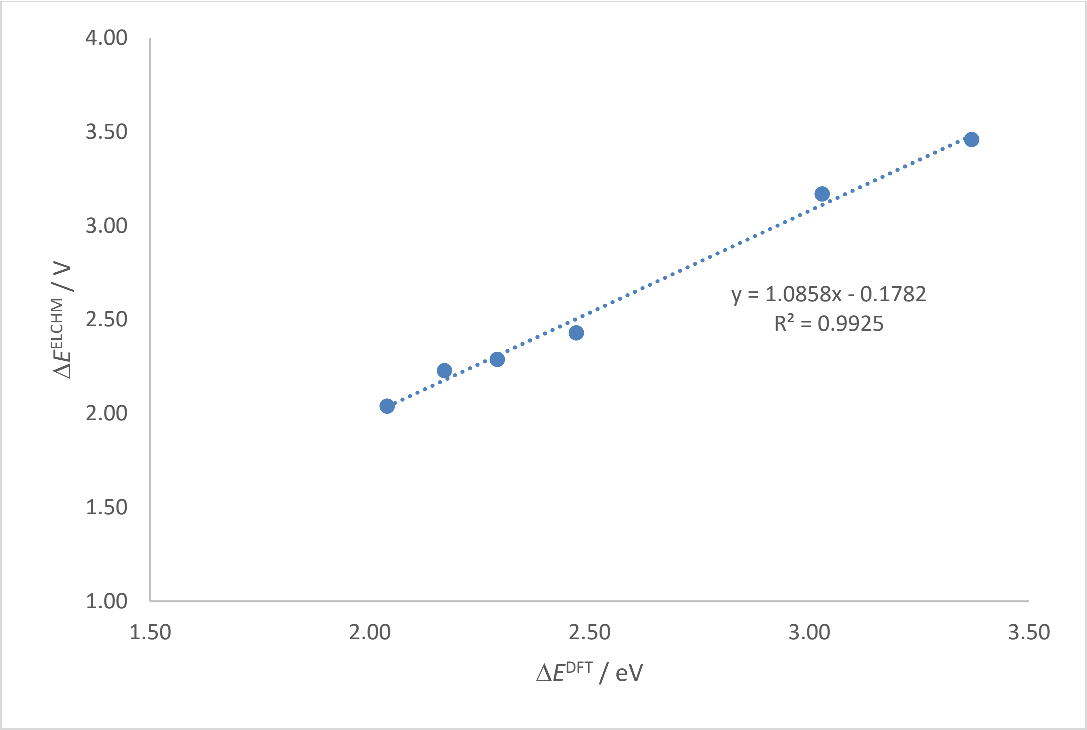

Figure S7: A correlation of the experimental (ELCHM) and DFT-calculated HOMO–LUMO gaps of chromophores **9–11**.

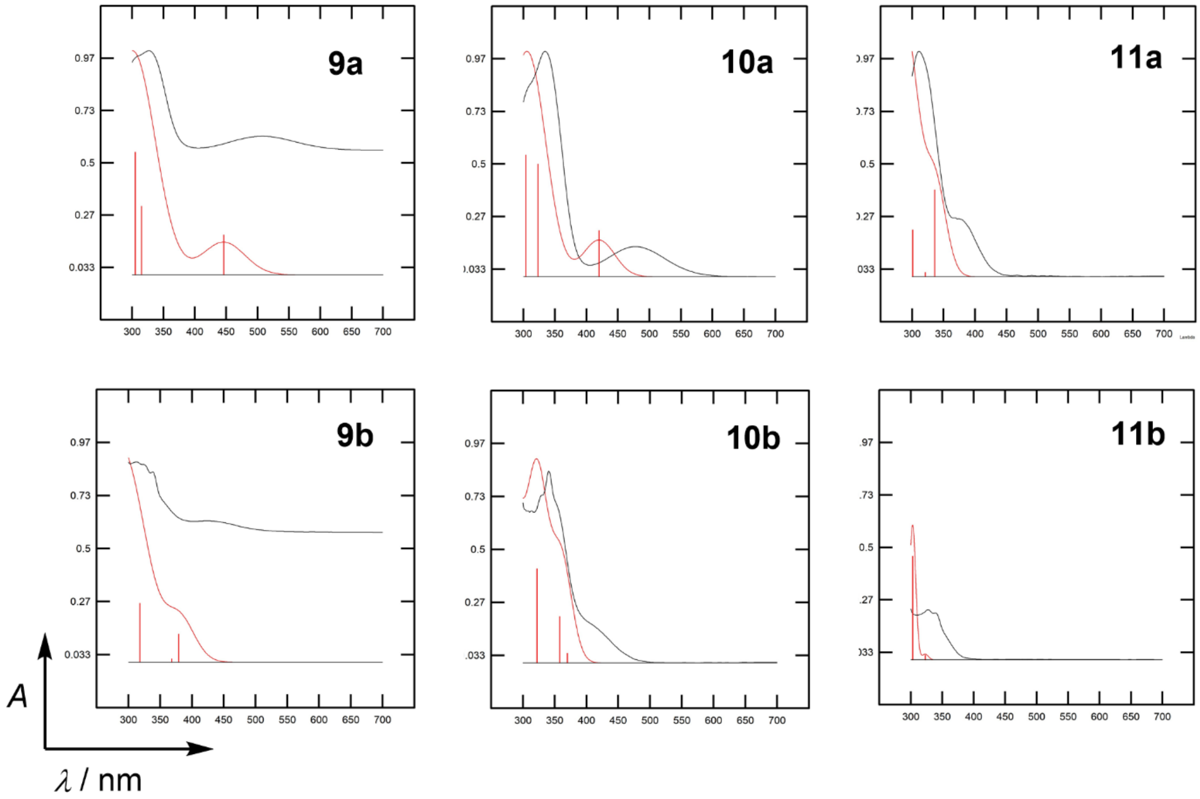

Figure S8: TD-DFT (n_{states} = 8) B3LYP/6-311+G(2d,f,p) calculated UV-Vis spectra of chromophores **1a**–**9a** in $CHCl_3$ (red curves) along with the experimental spectra (black curves). Red vertical lines represent oscillator strengths (*f*).

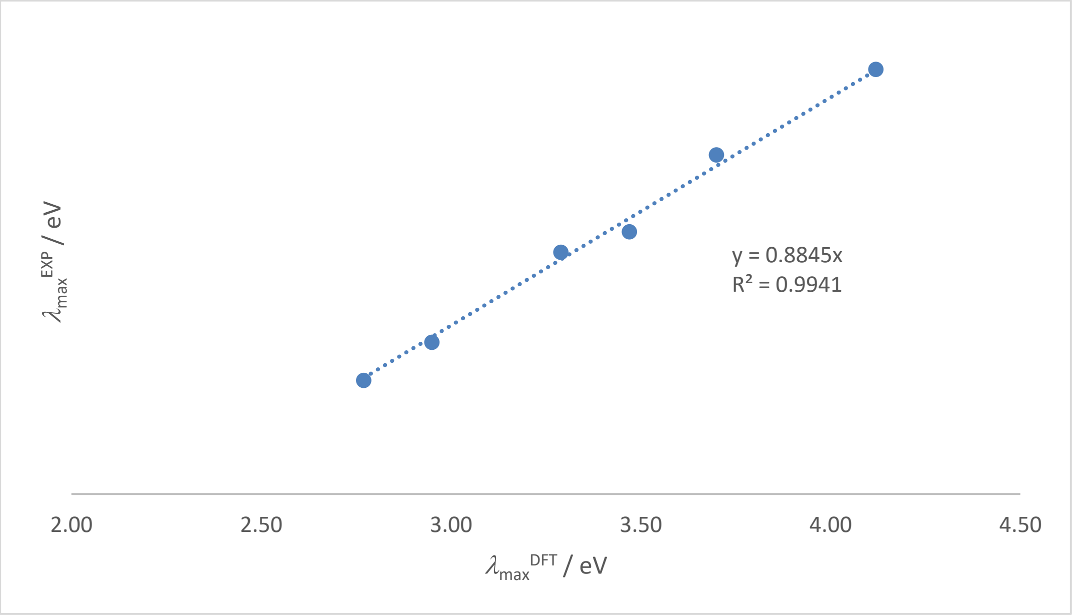

Figure S9: A correlation of the experimental (EXP) and DFT-calculated λ_{max} values of chromophores **9**–**11**.

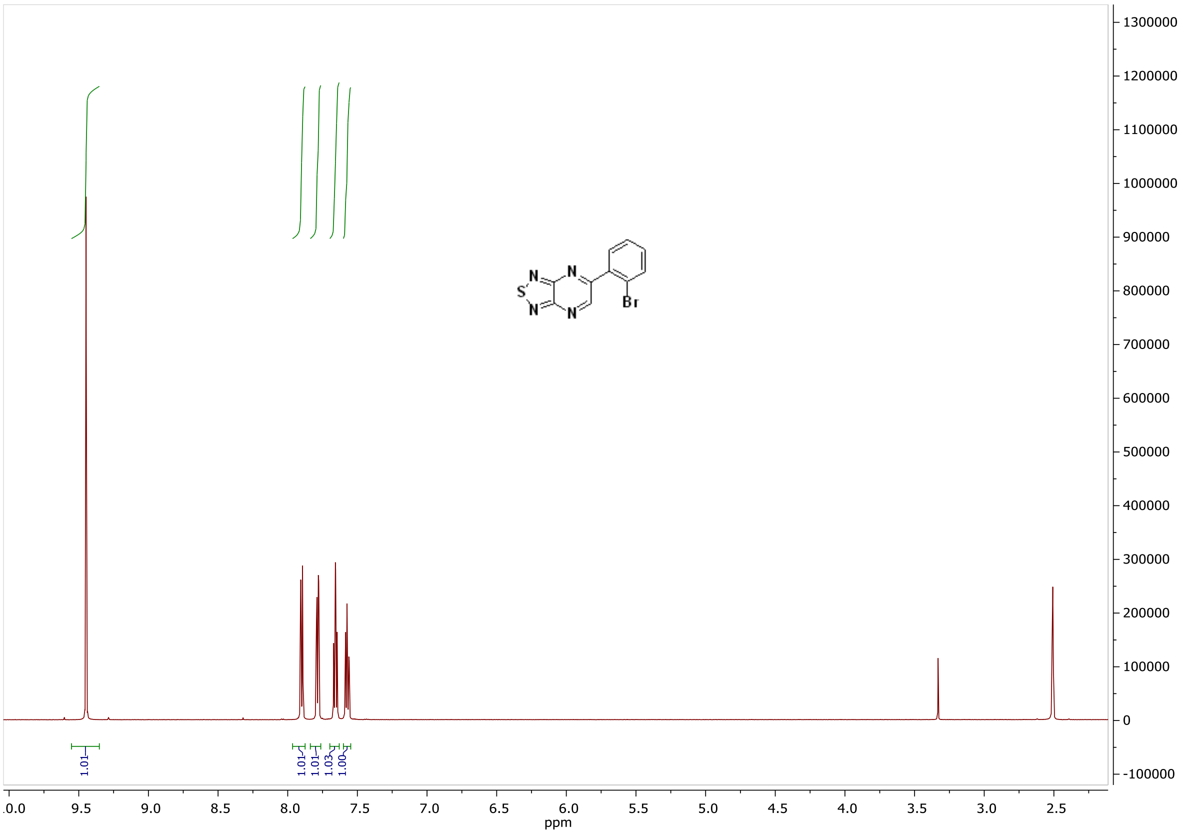

Figure S10. ^{1}H NMR (600 MHz, DMSO-d_6) spectrum of **6**.

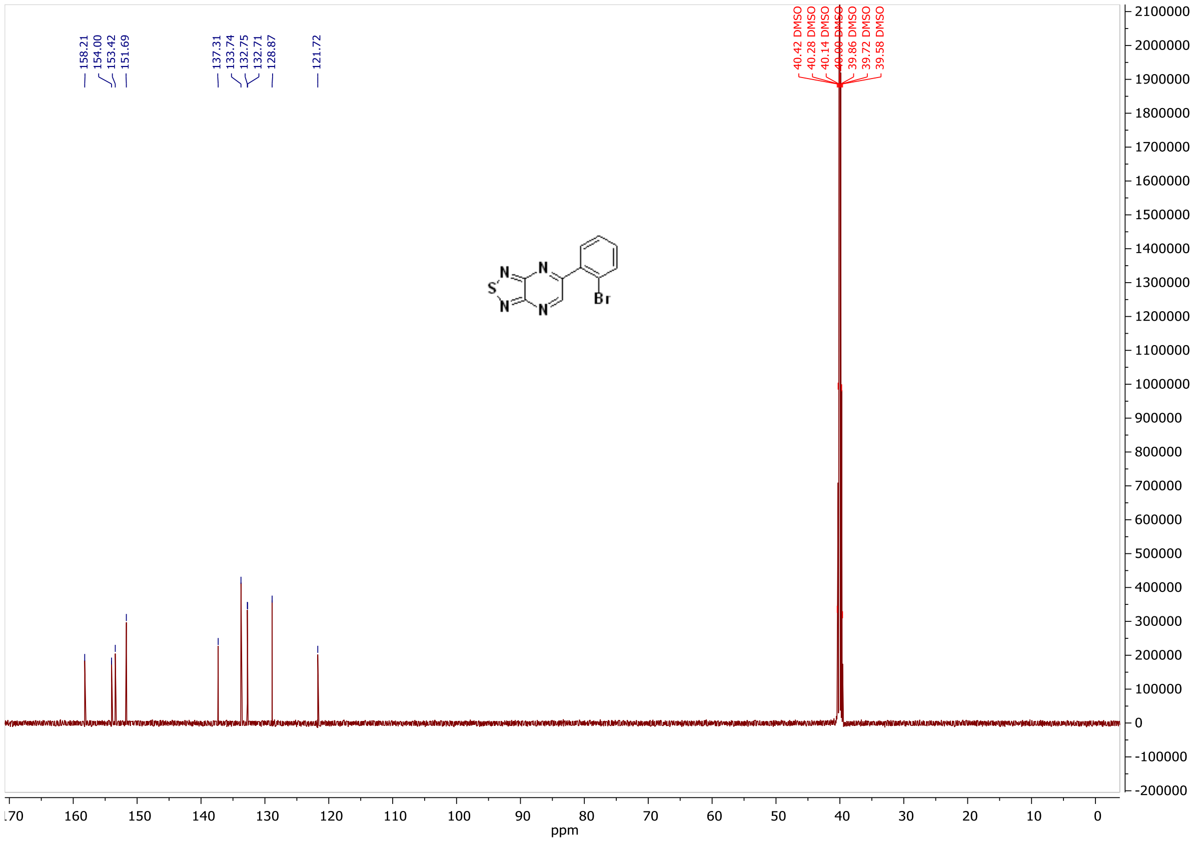

Figure S11. ^{13}C NMR (151 MHz, DMSO-d_6) spectrum of **6**.

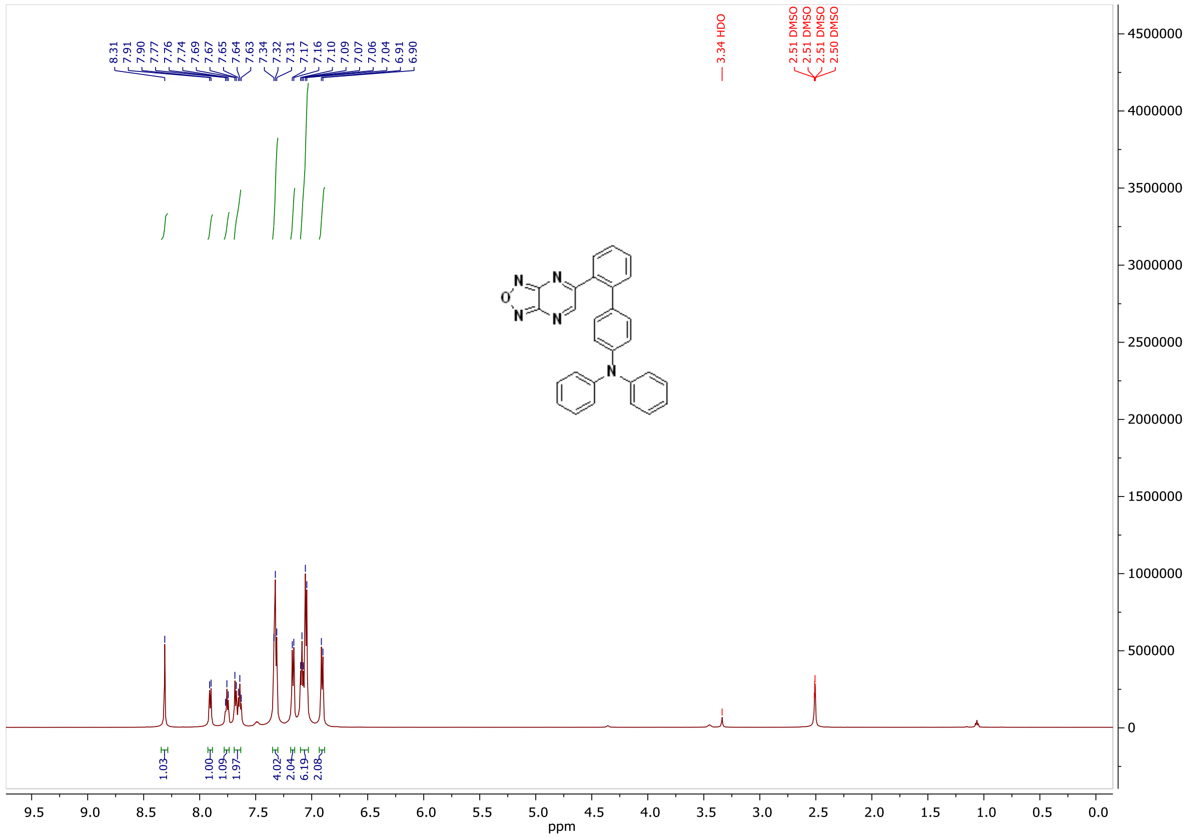

Figure S12. ^{1}H NMR (600 MHz, DMSO-d_6) spectrum of **9a**.

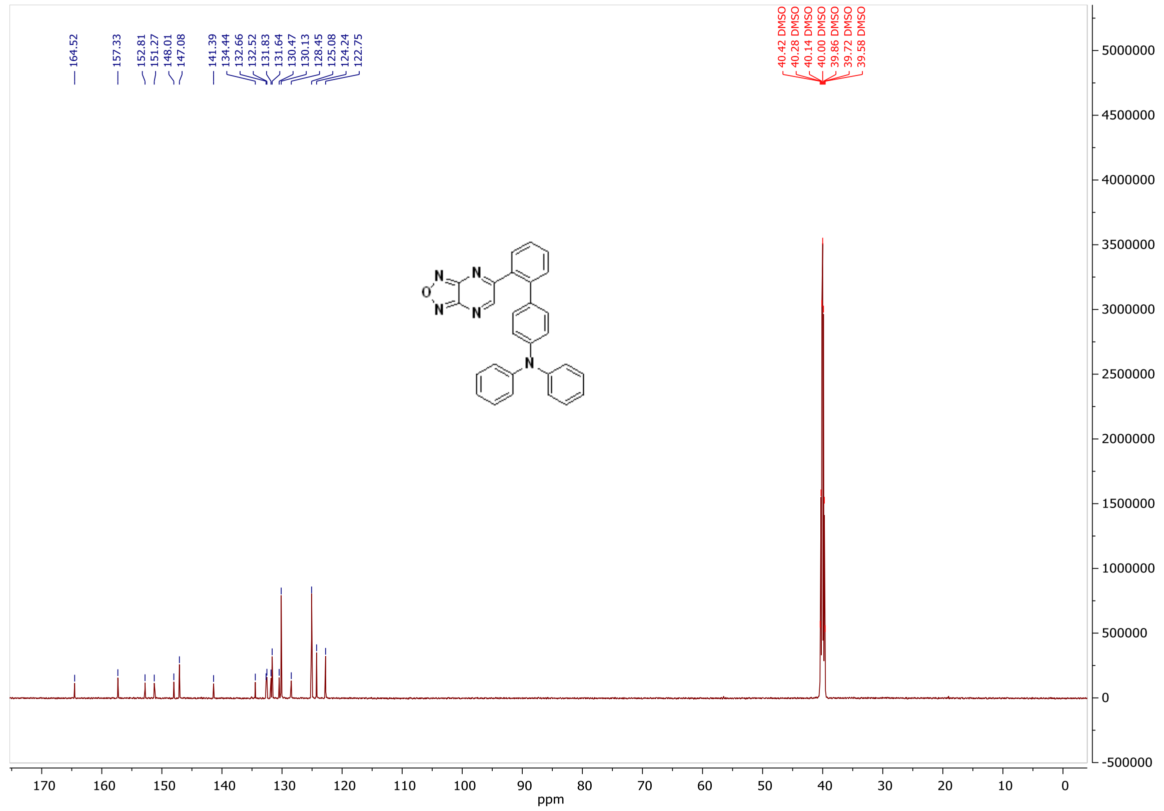

Figure S13. ^{13}C NMR (151 MHz, DMSO-d_6) spectrum of **9a**.

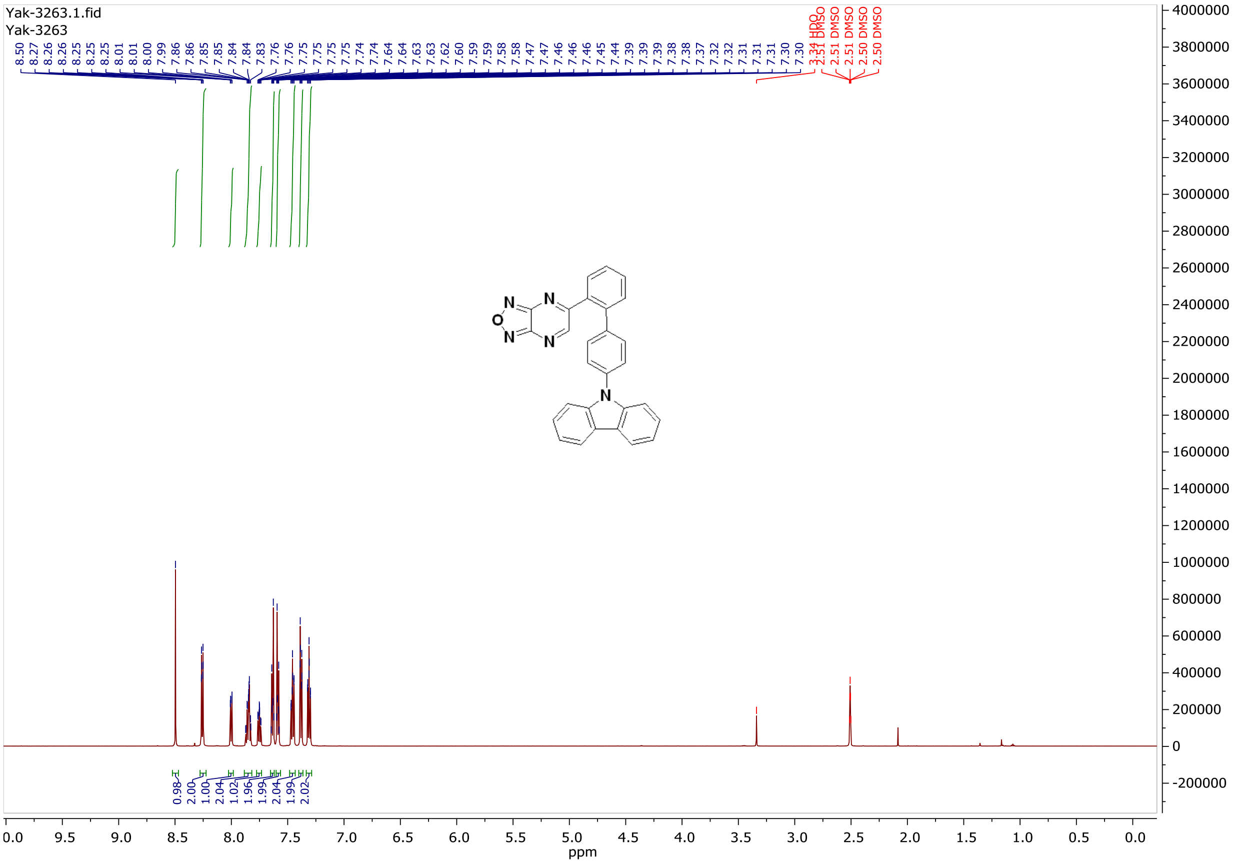

Figure S14. ^{1}H NMR (600 MHz, DMSO-d_6) spectrum of **9b**.

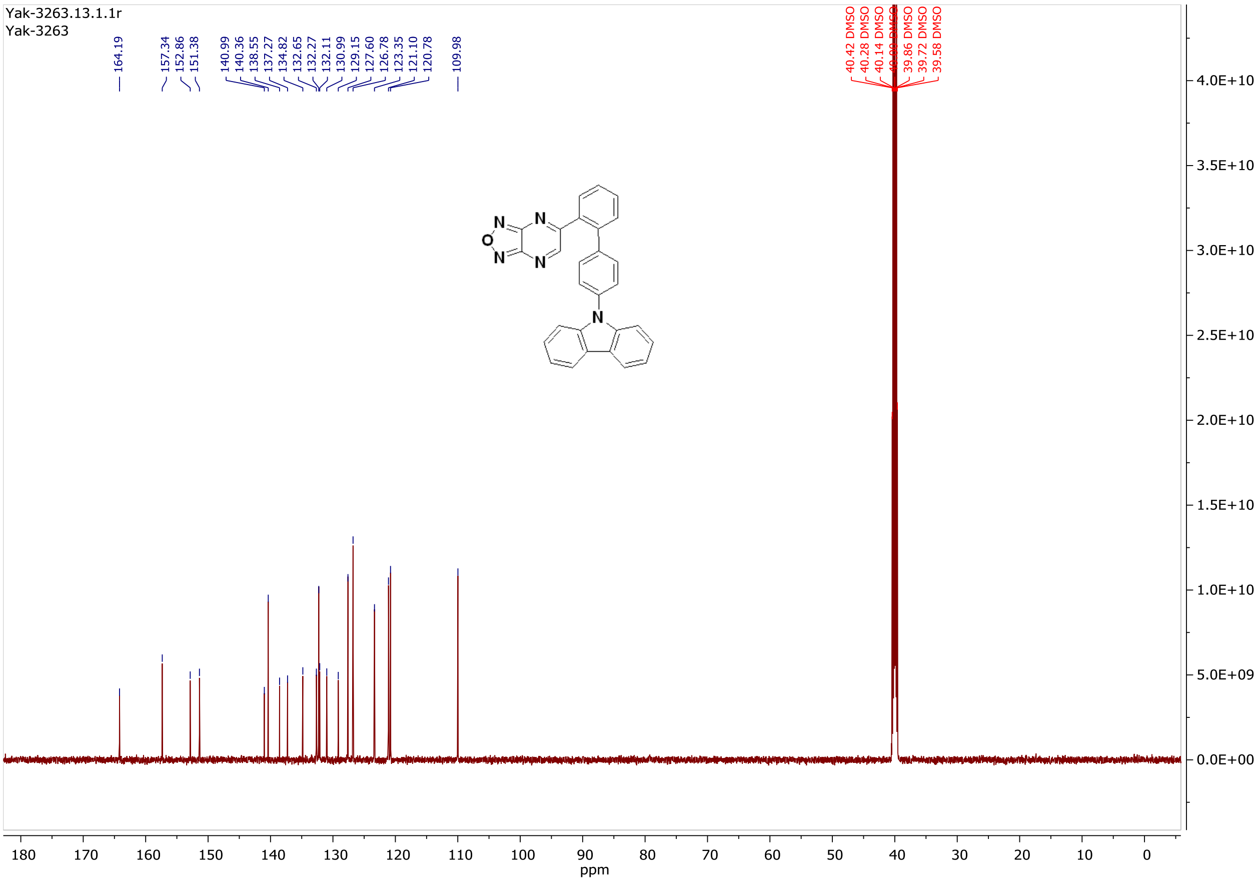

Figure S15. ^{13}C NMR (151 MHz, DMSO-d_6) spectrum of **9b**.

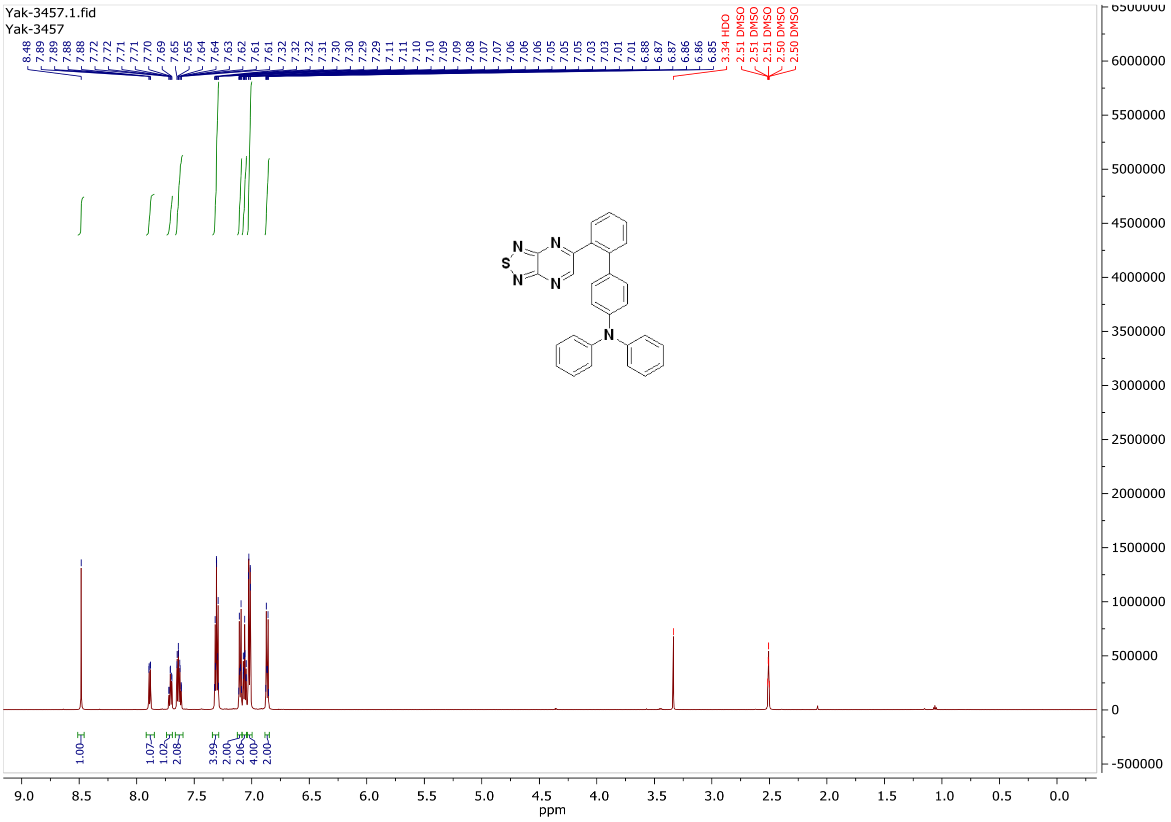

Figure S16. ^{1}H NMR (600 MHz, DMSO-d_6) spectrum of **10a**.

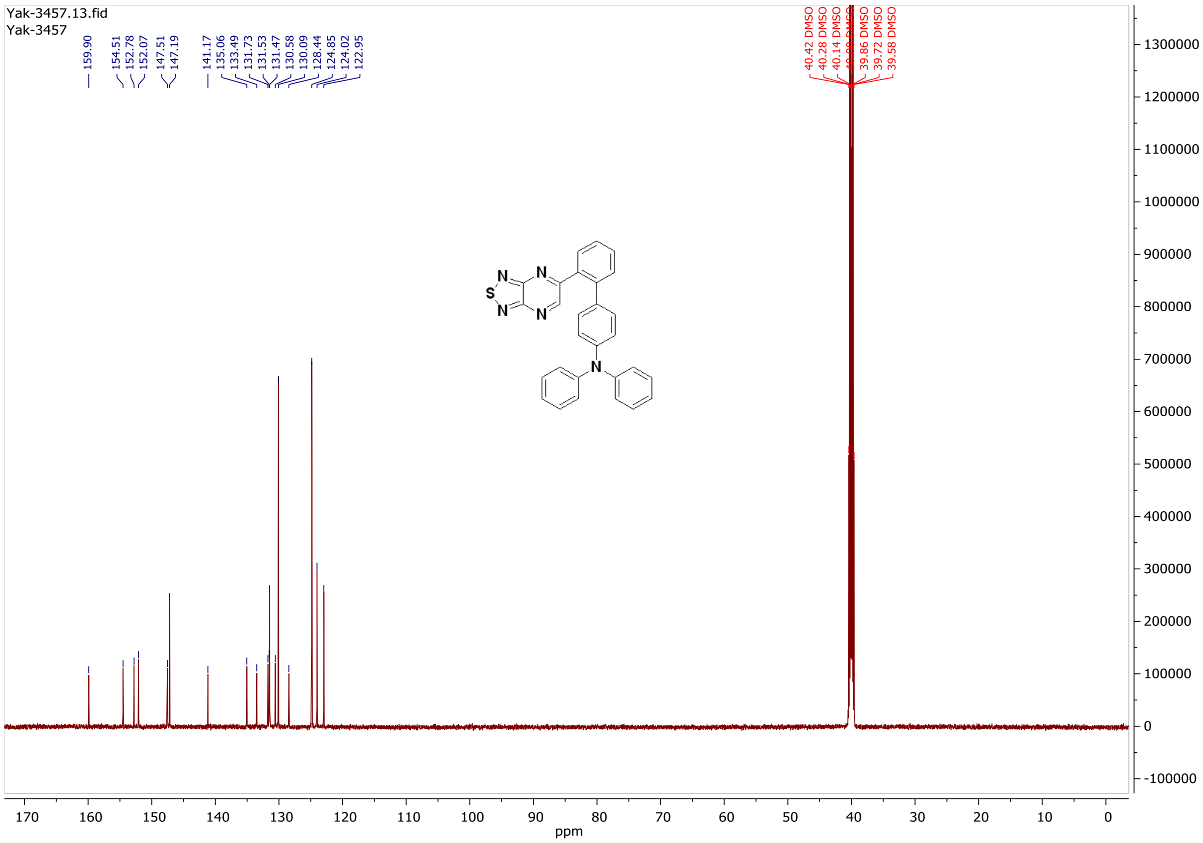

Figure S17. ^{13}C NMR (151 MHz, DMSO-d_6) spectrum of **10a**.

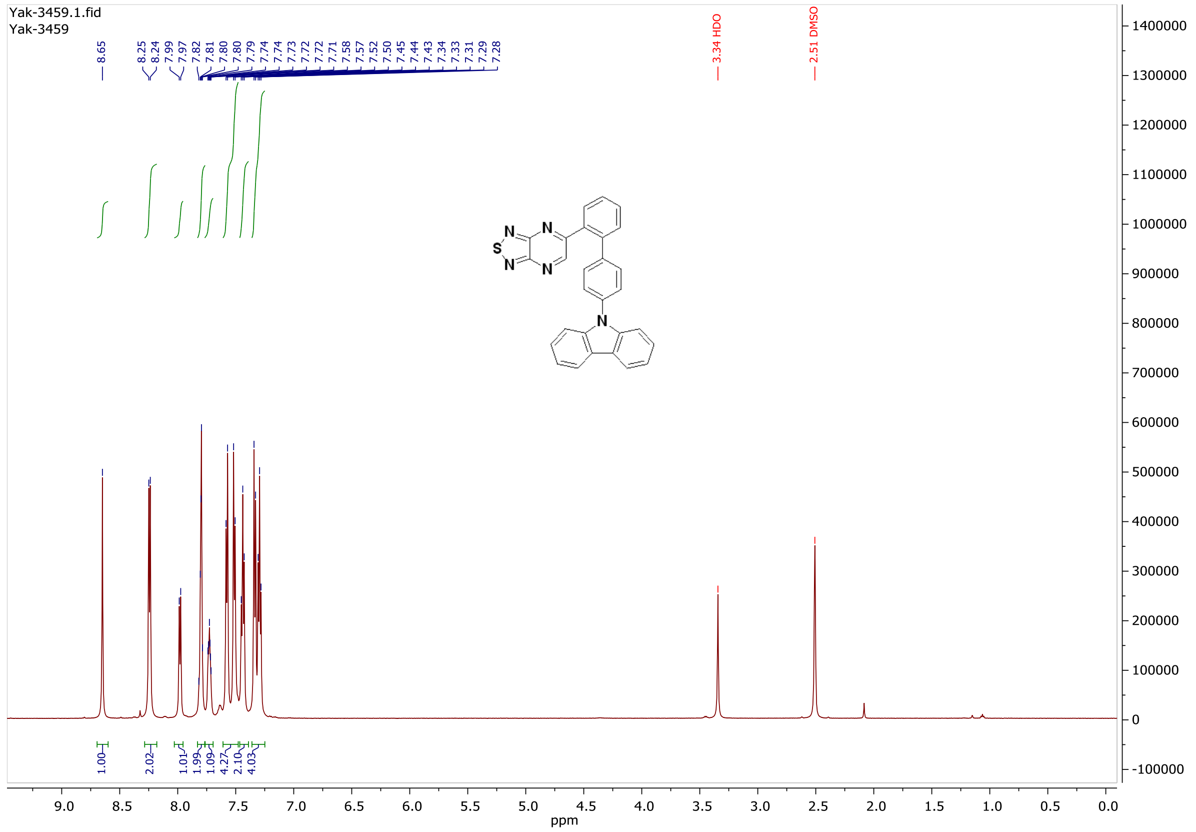

Figure S18. ^{1}H NMR (600 MHz, DMSO-d_6) spectrum of **10b**.

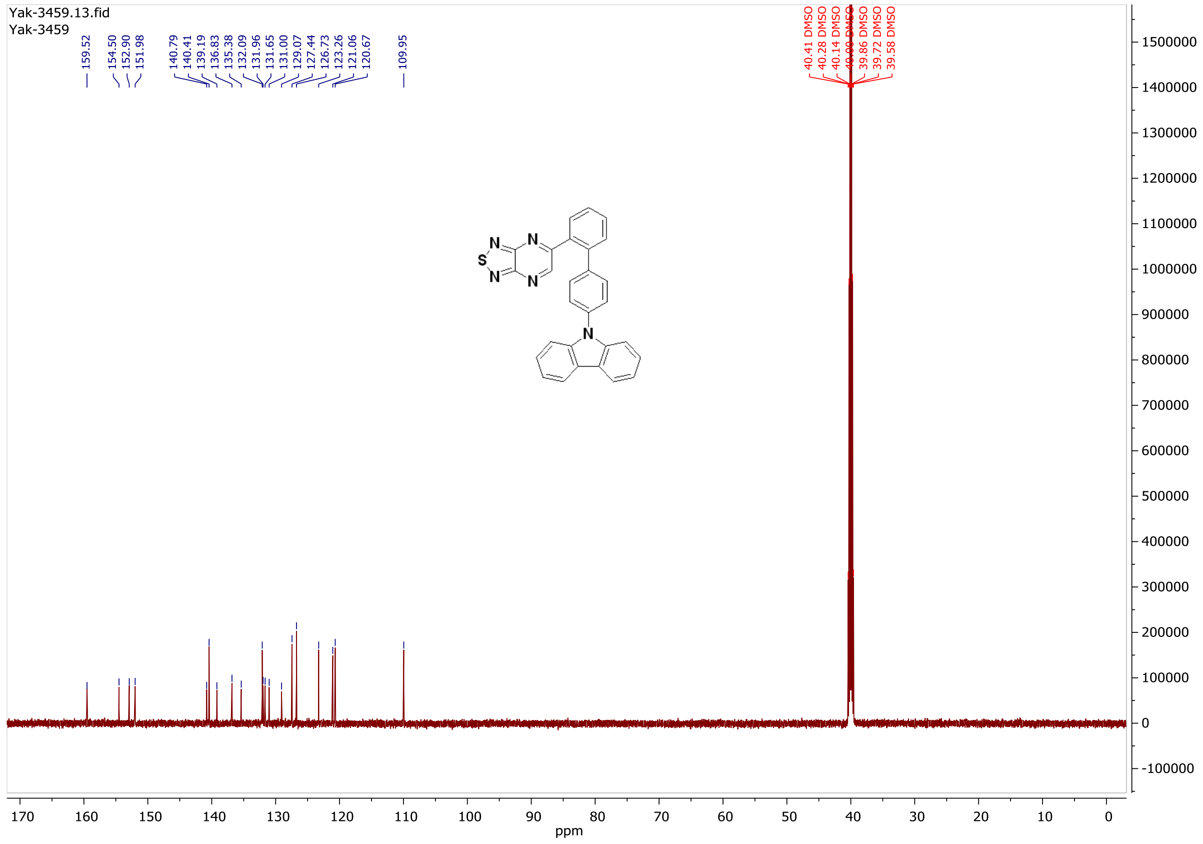

Figure S19. ^{13}C NMR (151 MHz, DMSO-d_6) spectrum of **10b**.

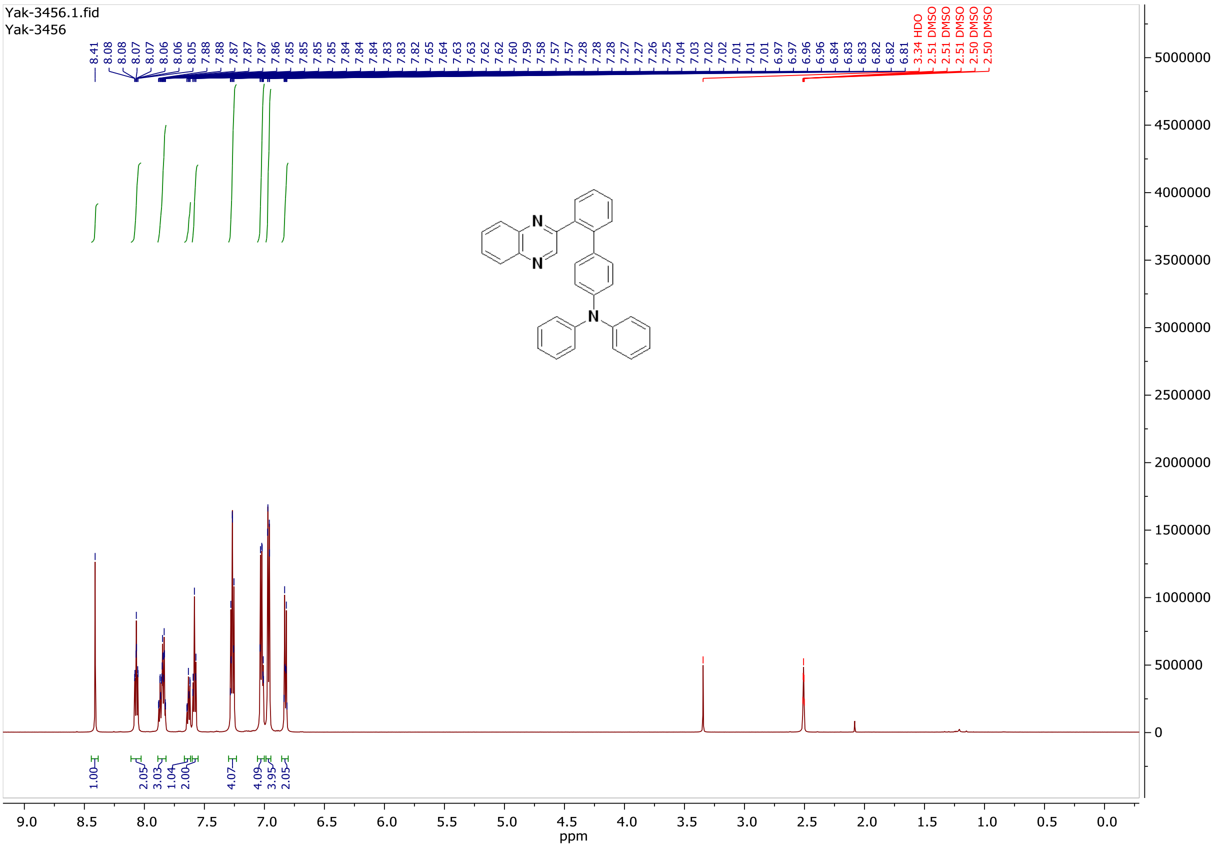

Figure S20. ^{1}H NMR (600 MHz, DMSO-d_6) spectrum of **11a**.

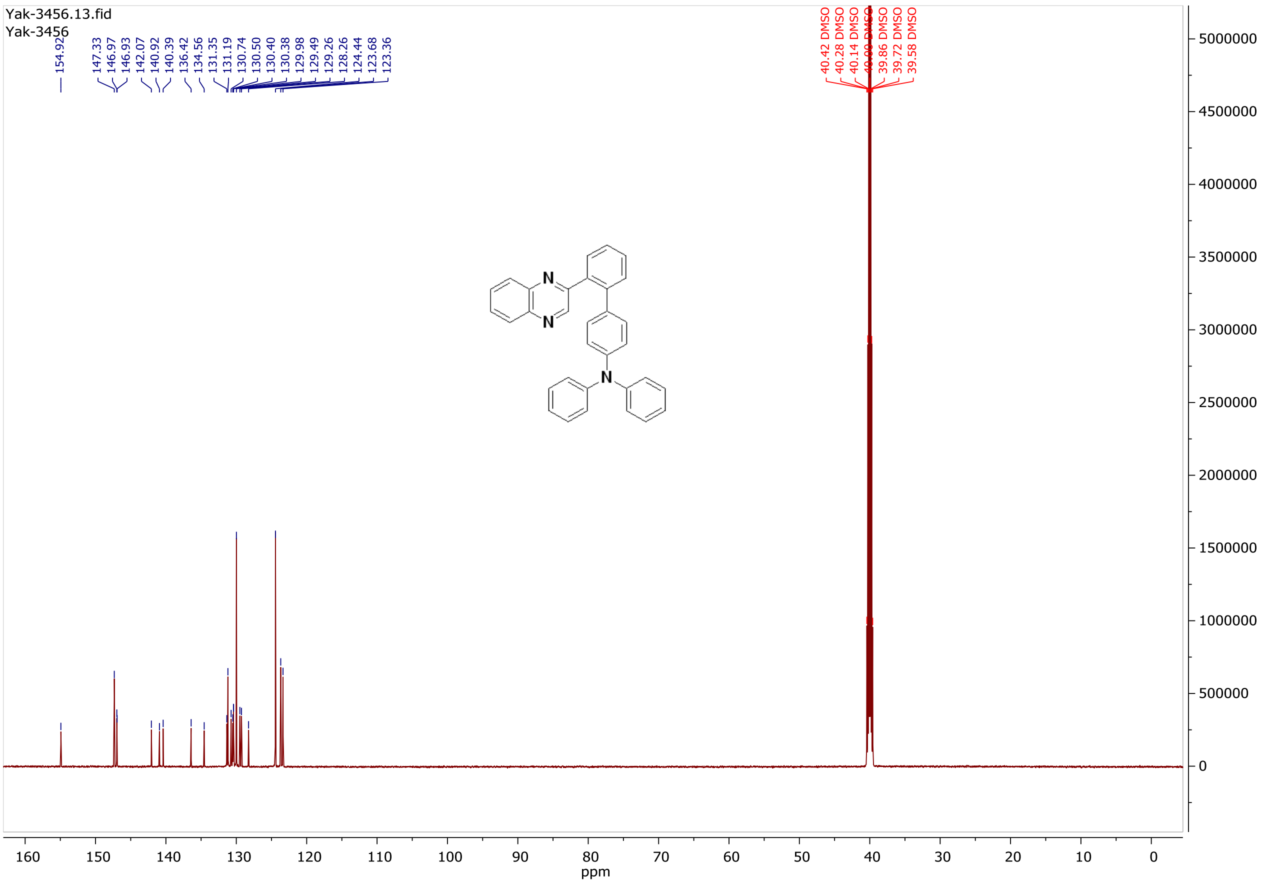

Figure S21. ^{13}C NMR (151 MHz, DMSO-d_6) spectrum of **11a**.

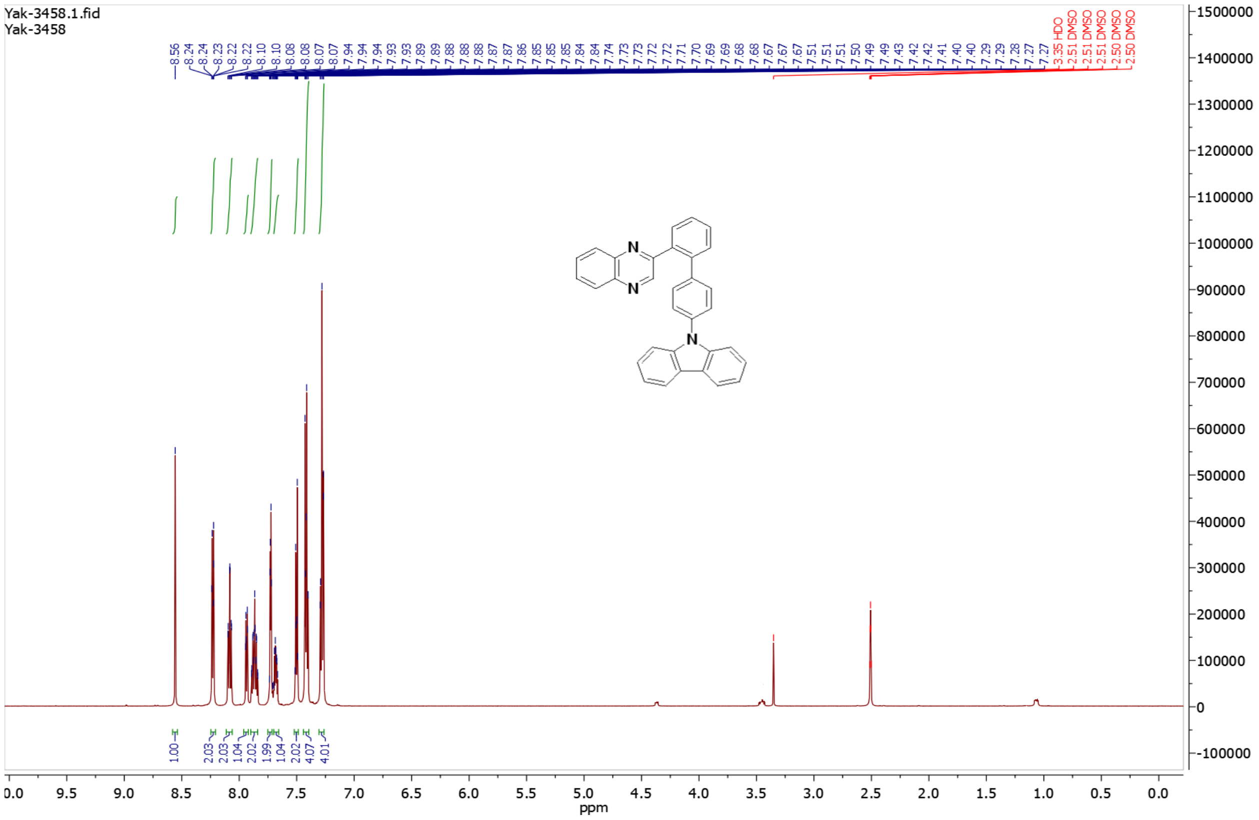

Figure S22. ^{1}H NMR (600 MHz, DMSO-d_6) spectrum of **11b**.

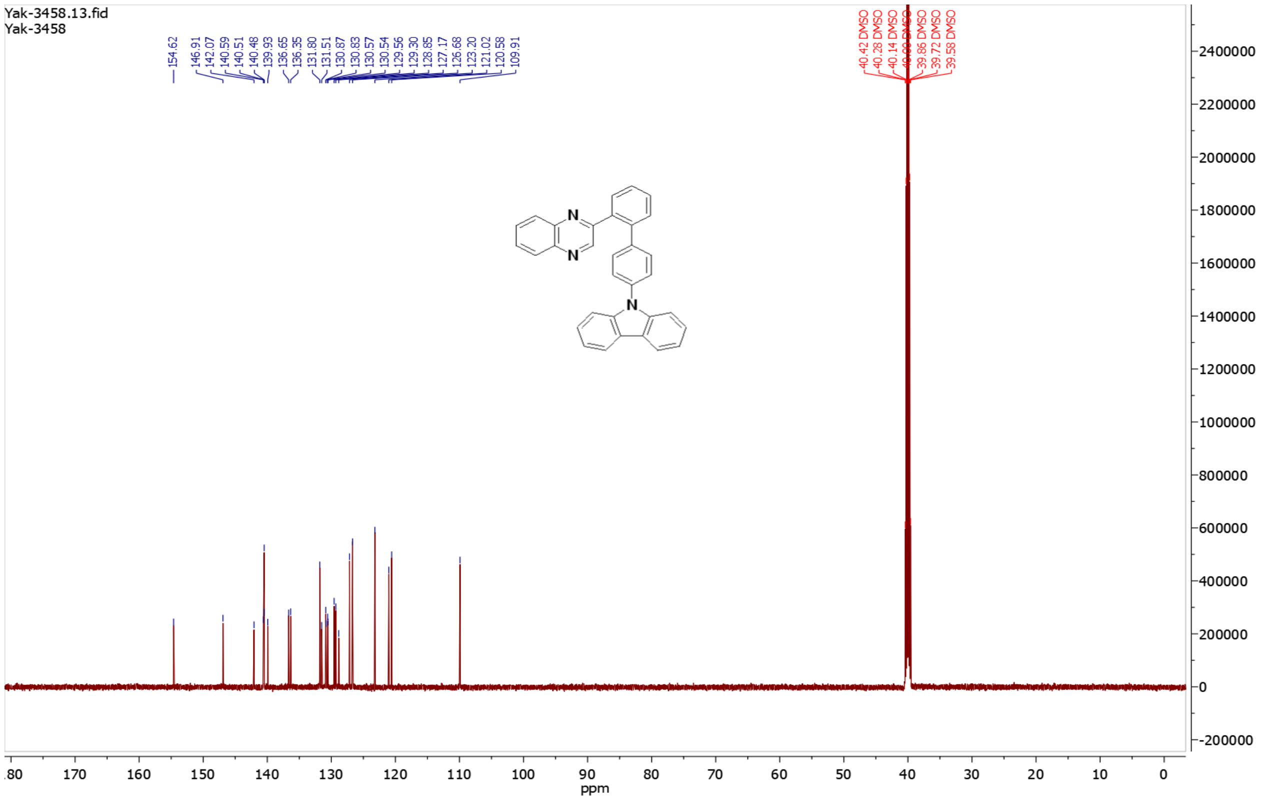

Figure S23. ^{13}C NMR (151 MHz, DMSO-d_6) spectrum of **11b**.

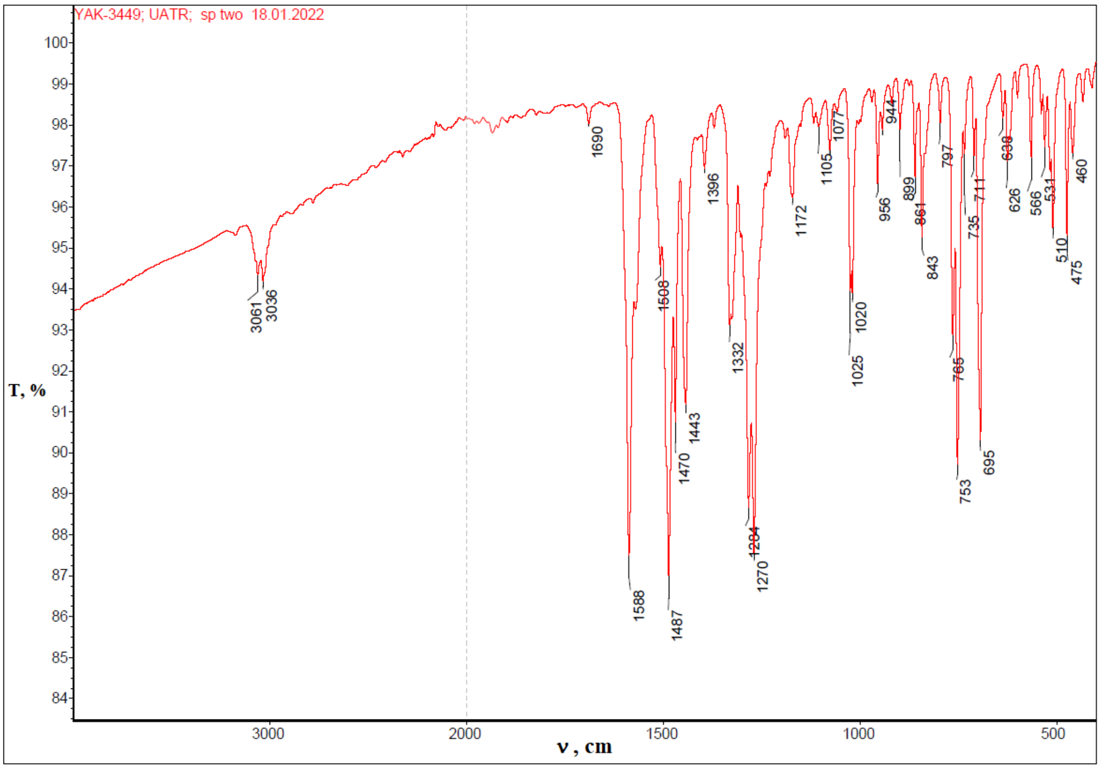

Figure S24. IR spectrum of **9a**.

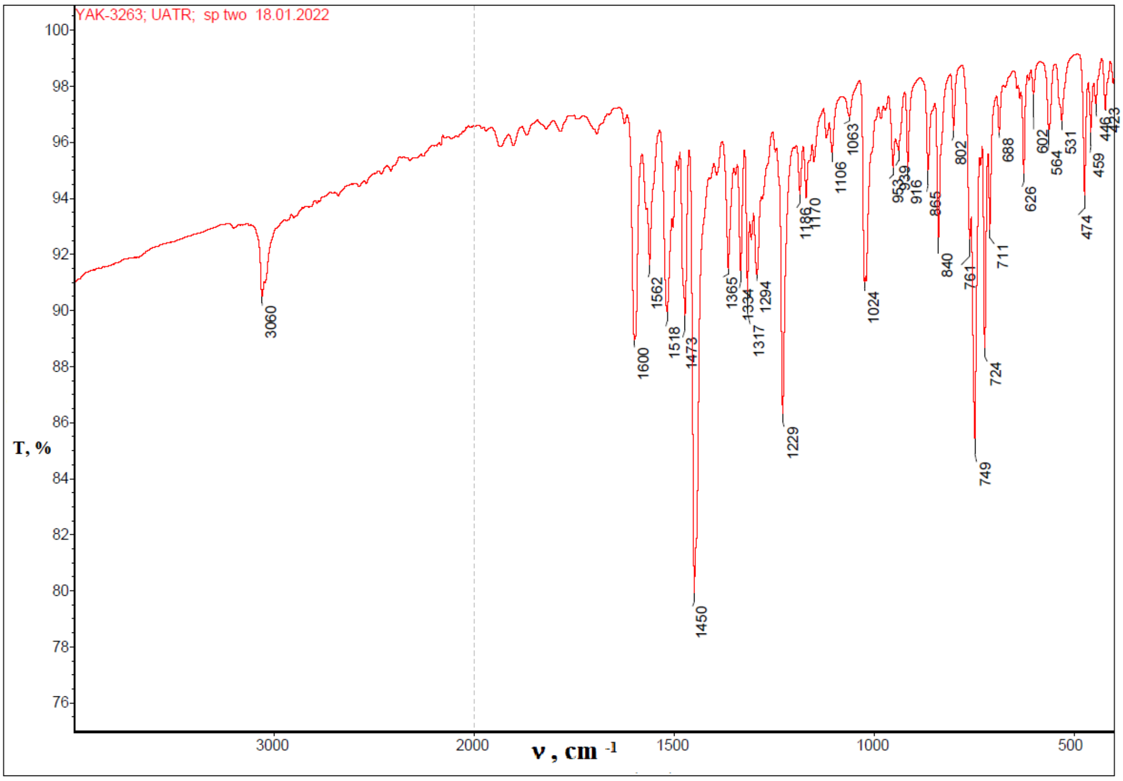

Figure S25. IR spectrum of **9b**.

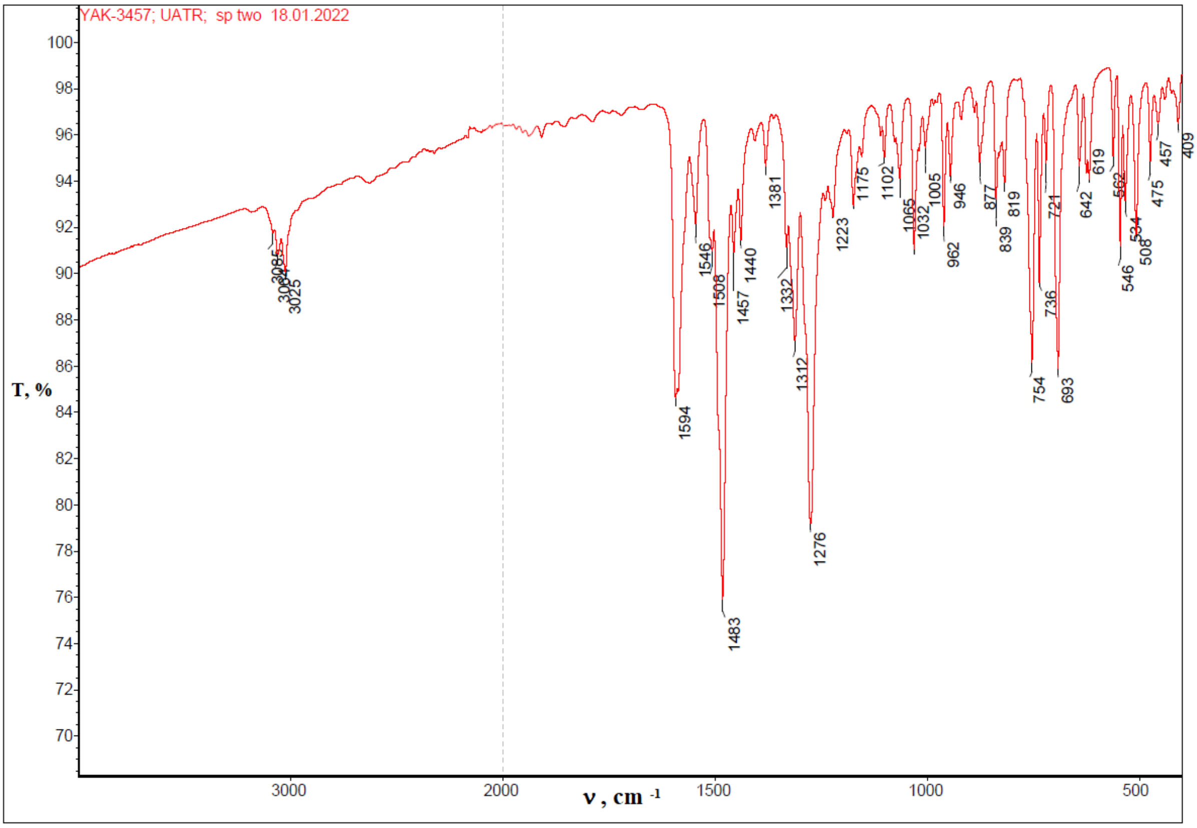

Figure S26. IR spectrum of **10a**.

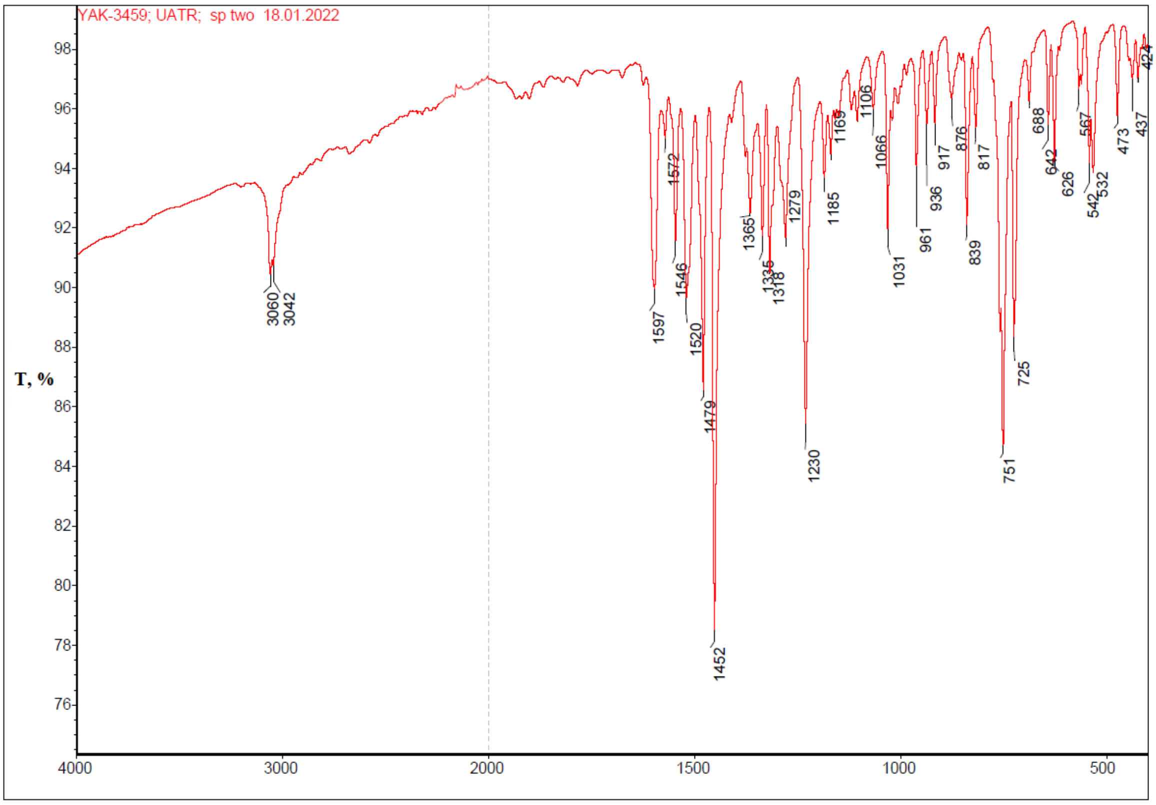

Figure S27. IR spectrum of **10b**.

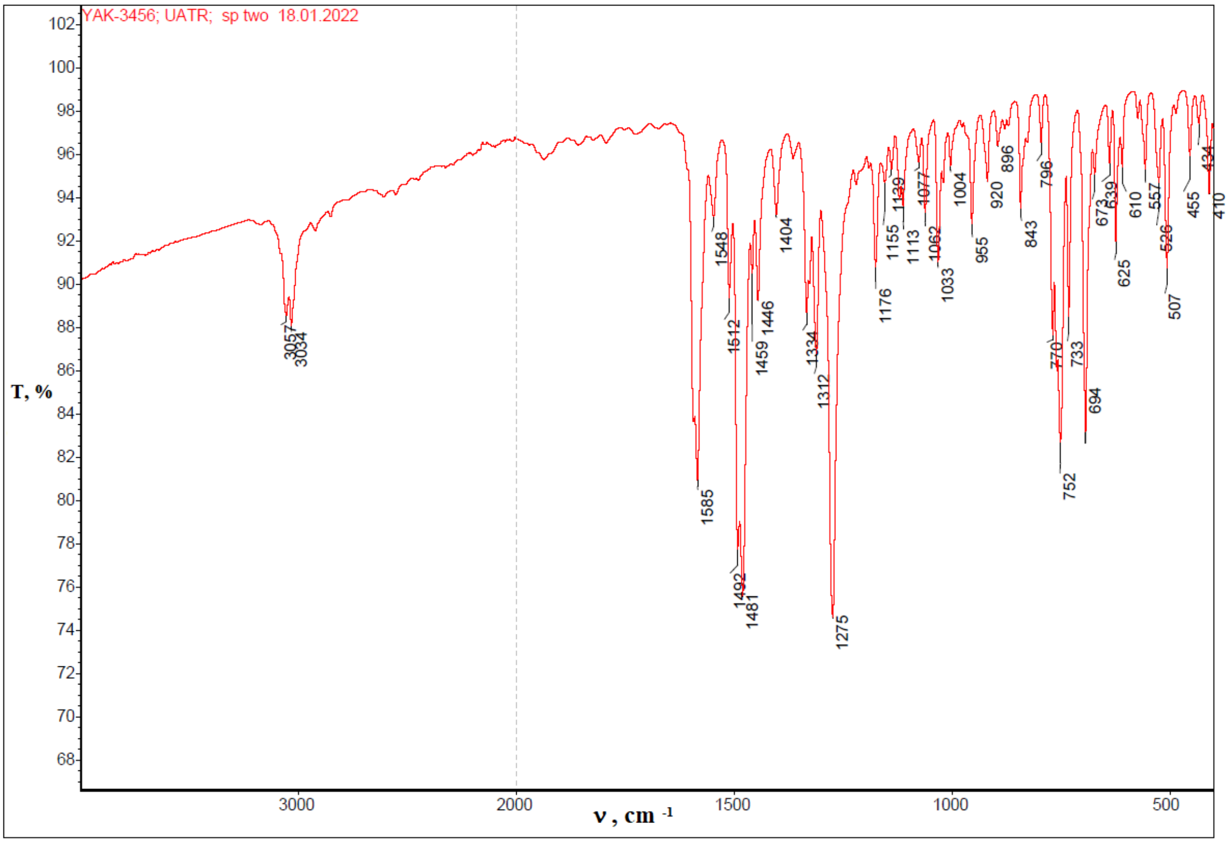

Figure S28. IR spectrum of **11a**.

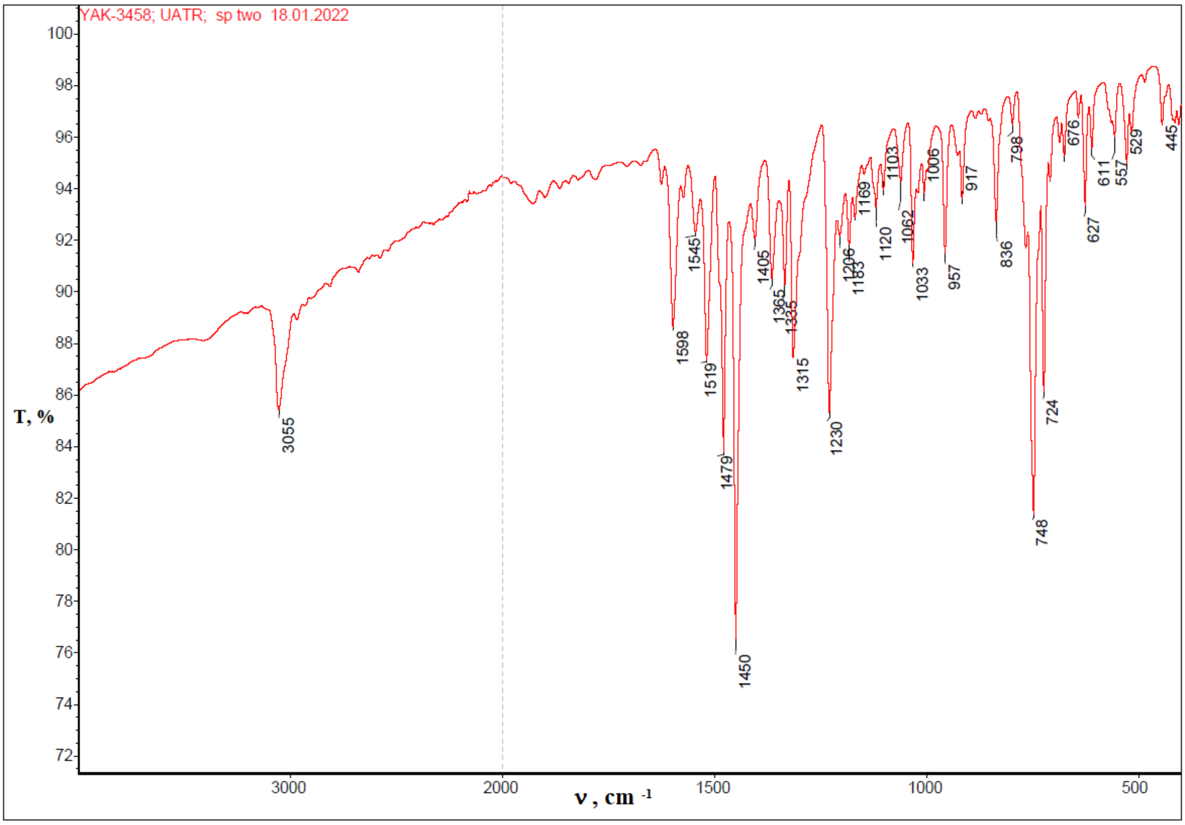

Figure S29. IR spectrum of **11b**.

TD-DFT data of chromophore 9a

```
Excitation energies and oscillator strengths
 iMO      E(MO)   Type MO
  111    -0.32320   HOMO-4
  112    -0.31824   HOMO-3
  113    -0.31720   HOMO-2
  114    -0.30659   HOMO-1
  115    -0.24746   HOMO
  116    -0.08610   LUMO
  117    -0.01380   LUMO+1
  118    -0.00078   LUMO+2
  119    0.00276   LUMO+3
  120    0.00995   LUMO+4

 EXCITED STATE   1:     SINGLET-A      2.7791 EV  446.12 NM  F=0.1775  <S**2>=0.000
     114 -> 116       -0.15570
     115 -> 116        0.67889
 THIS STATE FOR OPTIMIZATION AND/OR SECOND-ORDER CORRECTION.
 TOTAL ENERGY, E(TD-HF/TD-KS) =  -1426.88970916
 COPYING THE EXCITED STATE DENSITY FOR THIS STATE AS THE 1-PARTICLE RHOCI DENSITY.

 EXCITED STATE   2:     SINGLET-A      3.3705 EV  367.85 NM  F=0.0009  <S**2>=0.000
     107 -> 116        0.66695
     108 -> 116        0.14274

 EXCITED STATE   3:     SINGLET-A      3.9374 EV  314.89 NM  F=0.3059  <S**2>=0.000
     111 -> 116       -0.38269
     113 -> 116        0.20045
     114 -> 116        0.42922
     115 -> 116        0.13038
     115 -> 117        0.22254

 EXCITED STATE   4:     SINGLET-A      4.0613 EV  305.28 NM  F=0.5466  <S**2>=0.000
     111 -> 116        0.16971
     113 -> 116       -0.10625
     114 -> 116       -0.17415
```

115 -> 117 0.50776
115 -> 118 -0.29295
115 -> 119 -0.12272
115 -> 125 0.16204

EXCITED STATE 5: SINGLET-A 4.1883 EV 296.02 NM F=0.0985 <S**2>=0.000
115 -> 117 0.18267
115 -> 119 0.60721
115 -> 122 -0.14500

EXCITED STATE 6: SINGLET-A 4.2139 EV 294.23 NM F=0.0231 <S**2>=0.000
108 -> 116 -0.15451
111 -> 116 0.51864
114 -> 116 0.37936

EXCITED STATE 7: SINGLET-A 4.4197 EV 280.53 NM F=0.2841 <S**2>=0.000
115 -> 120 -0.22000
115 -> 121 0.61242
115 -> 122 0.15444

EXCITED STATE 8: SINGLET-A 4.5013 EV 275.44 NM F=0.0443 <S**2>=0.000
107 -> 116 -0.10202
109 -> 116 0.45099
112 -> 116 0.36153
113 -> 116 0.29349

TD-DFT data of chromophore 9b

Excitation energies and oscillator strengths

iMO	E(MO)	Type MO
110	-0.32615	HOMO-4
111	-0.32148	HOMO-3
112	-0.30854	HOMO-2
113	-0.27624	HOMO-1
114	-0.26169	HOMO
115	-0.08898	LUMO
116	-0.01781	LUMO+1
117	-0.00563	LUMO+2
118	-0.00411	LUMO+3
119	0.00474	LUMO+4

EXCITED STATE 1: SINGLET-A 3.2678 EV 379.41 NM F=0.1231 <S**2>=0.000
107 -> 115 0.23693
108 -> 115 0.10678
112 -> 115 0.26105
114 -> 115 0.58960
THIS STATE FOR OPTIMIZATION AND/OR SECOND-ORDER CORRECTION.
TOTAL ENERGY, E(TD-HF/TD-KS) = -1425.69576463
COPYING THE EXCITED STATE DENSITY FOR THIS STATE AS THE 1-PARTICLE RHOCI DENSITY.

EXCITED STATE 2: SINGLET-A 3.3689 EV 368.03 NM F=0.0141 <S**2>=0.000
107 -> 115 0.56184
108 -> 115 0.28065
114 -> 115 -0.28138

EXCITED STATE 3: SINGLET-A 3.8954 EV 318.28 NM F=0.2590 <S**2>=0.000
107 -> 115 -0.10193
110 -> 115 0.32204
112 -> 115 0.55496
114 -> 115 -0.22845

EXCITED STATE 4: SINGLET-A 3.9614 EV 312.98 NM F=0.0006 <S**2>=0.000
113 -> 115 0.70336

EXCITED STATE 5: SINGLET-A 4.1646 EV 297.71 NM F=0.0736 <S**2>=0.000
110 -> 115 0.60079
112 -> 115 -0.29724

EXCITED STATE 6: SINGLET-A 4.2706 EV 290.32 NM F=0.0887 <S**2>=0.000
113 -> 127 0.17996
114 -> 117 0.65584

EXCITED STATE 7: SINGLET-A 4.4863 EV 276.36 NM F=0.3571 <S**2>=0.000
109 -> 115 0.10652
112 -> 116 0.15322
114 -> 116 0.56076
114 -> 118 -0.25256
114 -> 124 -0.16940

EXCITED STATE 8: SINGLET-A 4.6324 EV 267.65 NM F=0.0310 <S**2>=0.000
105 -> 115 -0.14259
107 -> 115 0.12661
109 -> 115 0.55214
111 -> 115 -0.19927
113 -> 117 0.16382
114 -> 116 -0.10247
114 -> 119 0.13119

TD-DFT data of chromophore 10a

Excitation energies and oscillator strengths

iMO	E(MO)	Type MO
115	-0.31742	HOMO-4
116	-0.31633	HOMO-3
117	-0.31324	HOMO-2
118	-0.30109	HOMO-1
119	-0.24543	HOMO
120	-0.08055	LUMO
121	-0.01049	LUMO+1
122	0.00173	LUMO+2
123	0.00445	LUMO+3
124	0.01065	LUMO+4

EXCITED STATE 1: SINGLET-A 2.9515 EV 420.07 NM F=0.2035 <S**2>=0.000
118 -> 120 -0.17422
119 -> 120 0.67171
THIS STATE FOR OPTIMIZATION AND/OR SECOND-ORDER CORRECTION.
TOTAL ENERGY, E(TD-HF/TD-KS) = -1749.95757881
COPYING THE EXCITED STATE DENSITY FOR THIS STATE AS THE 1-PARTICLE RHOCI DENSITY.

EXCITED STATE 2: SINGLET-A 3.3779 EV 367.04 NM F=0.0007 <S**2>=0.000
112 -> 120 0.67003
113 -> 120 0.10667

EXCITED STATE 3: SINGLET-A 3.8427 EV 322.65 NM F=0.4993 <S**2>=0.000
110 -> 120 -0.11866
115 -> 120 -0.15756
117 -> 120 0.35168
118 -> 120 0.52165
119 -> 120 0.14957

EXCITED STATE 4: SINGLET-A 4.0769 EV 304.12 NM F=0.5395 <S**2>=0.000
119 -> 121 0.51388

```
    119 -> 122       -0.37329
    119 -> 129       -0.16107

 EXCITED STATE   5:      SINGLET-A      4.1751 EV  296.96 NM  F=0.1127  <S**2>=0.000
    119 -> 121        0.23114
    119 -> 122        0.24181
    119 -> 123        0.53153
    119 -> 126       -0.18892

 EXCITED STATE   6:      SINGLET-A      4.3669 EV  283.92 NM  F=0.0216  <S**2>=0.000
    111 -> 120        0.15295
    115 -> 120        0.37980
    116 -> 120        0.14603
    117 -> 120       -0.33703
    118 -> 120        0.32849
    119 -> 122        0.10257

 EXCITED STATE   7:      SINGLET-A      4.3883 EV  282.54 NM  F=0.2781  <S**2>=0.000
    119 -> 124        0.13775
    119 -> 125        0.62636
    119 -> 126       -0.16442

 EXCITED STATE   8:      SINGLET-A      4.5693 EV  271.34 NM  F=0.0482  <S**2>=0.000
    107 -> 120        0.15314
    109 -> 120        0.15797
    110 -> 120        0.42144
    111 -> 120        0.14595
    113 -> 120       -0.30210
    116 -> 120       -0.29070
```

TD-DFT data of chromophore 10b

Excitation energies and oscillator strengths

```
 iMO      E(MO)    Type MO
  114    -0.32034   HOMO-4
  115    -0.31750   HOMO-3
  116    -0.30199   HOMO-2
  117    -0.27521   HOMO-1
  118    -0.26049   HOMO
  119    -0.08289   LUMO
  120    -0.01419   LUMO+1
  121    -0.00473   LUMO+2
  122    -0.00082   LUMO+3
  123     0.00614   LUMO+4

 EXCITED STATE   1:      SINGLET-A      3.3535 EV  369.71 NM  F=0.0424  <S**2>=0.000
    112 -> 119        0.61766
    113 -> 119        0.13219
    116 -> 119       -0.18512
    118 -> 119        0.20519
 THIS STATE FOR OPTIMIZATION AND/OR SECOND-ORDER CORRECTION.
 TOTAL ENERGY, E(TD-HF/TD-KS) =  -1748.76721752
 COPYING THE EXCITED STATE DENSITY FOR THIS STATE AS THE 1-PARTICLE RHOCI DENSITY.

 EXCITED STATE   2:      SINGLET-A      3.4623 EV  358.10 NM  F=0.2038  <S**2>=0.000
    112 -> 119       -0.25383
    116 -> 119       -0.27643
    118 -> 119        0.57481

 EXCITED STATE   3:      SINGLET-A      3.8451 EV  322.45 NM  F=0.4142  <S**2>=0.000
    115 -> 119       -0.34854
    116 -> 119        0.49742
    118 -> 119        0.31398
```

EXCITED STATE 4: SINGLET-A 4.1363 EV 299.75 NM F=0.0002 <S**2>=0.000
117 -> 119 0.70358

EXCITED STATE 5: SINGLET-A 4.2628 EV 290.85 NM F=0.0921 <S**2>=0.000
117 -> 131 0.18142
118 -> 121 0.65707

EXCITED STATE 6: SINGLET-A 4.3050 EV 288.00 NM F=0.0505 <S**2>=0.000
115 -> 119 0.55984
115 -> 120 -0.10942
116 -> 119 0.32081
118 -> 119 0.11995

EXCITED STATE 7: SINGLET-A 4.5427 EV 272.93 NM F=0.3596 <S**2>=0.000
113 -> 119 -0.10302
116 -> 120 -0.15425
118 -> 120 0.54810
118 -> 122 0.26779
118 -> 128 0.11868
118 -> 129 -0.13155

EXCITED STATE 8: SINGLET-A 4.5917 EV 270.02 NM F=0.0384 <S**2>=0.000
107 -> 119 -0.35206
108 -> 119 0.15663
110 -> 119 0.42417
111 -> 119 0.17300
113 -> 119 -0.26048

TD-DFT data of chromophore 11a

Excitation energies and oscillator strengths

iMO	E(MO)	Type MO
114	-0.31440	HOMO-4
115	-0.31036	HOMO-3
116	-0.30543	HOMO-2
117	-0.29317	HOMO-1
118	-0.24334	HOMO
119	-0.04364	LUMO
120	-0.00627	LUMO+1
121	0.00223	LUMO+2
122	0.00650	LUMO+3
123	0.01275	LUMO+4

EXCITED STATE 1: SINGLET-A 3.6920 EV 335.82 NM F=0.3842 <S**2>=0.000
112 -> 119 0.11394
117 -> 119 -0.23894
118 -> 119 0.58664
118 -> 120 -0.13530
118 -> 121 -0.12700
118 -> 122 -0.13210
THIS STATE FOR OPTIMIZATION AND/OR SECOND-ORDER CORRECTION.
TOTAL ENERGY, E(TD-HF/TD-KS) = -1397.03413784
COPYING THE EXCITED STATE DENSITY FOR THIS STATE AS THE 1-PARTICLE RHOCI DENSITY.

EXCITED STATE 2: SINGLET-A 3.8569 EV 321.46 NM F=0.0181 <S**2>=0.000
110 -> 119 -0.16819
112 -> 119 0.61871
114 -> 119 0.11016
118 -> 119 -0.12808

EXCITED STATE 3: SINGLET-A 4.1149 EV 301.31 NM F=0.2068 <S**2>=0.000
118 -> 120 -0.29300

```
   118 -> 121       -0.38593
   118 -> 122        0.42121
   118 -> 123       -0.13358

 EXCITED STATE   4:      SINGLET-A      4.2382 EV  292.54 NM  F=0.3699  <S**2>=0.000
   115 -> 119       -0.12516
   117 -> 119        0.39336
   118 -> 120       -0.25142
   118 -> 121       -0.21128
   118 -> 122       -0.34557

 EXCITED STATE   5:      SINGLET-A      4.3550 EV  284.69 NM  F=0.3038  <S**2>=0.000
   118 -> 123       -0.36696
   118 -> 124        0.55994

 EXCITED STATE   6:      SINGLET-A      4.3610 EV  284.30 NM  F=0.1348  <S**2>=0.000
   109 -> 119       -0.10586
   115 -> 119       -0.12732
   116 -> 119       -0.21380
   117 -> 119        0.38218
   118 -> 119        0.30508
   118 -> 120        0.16452
   118 -> 121        0.12430
   118 -> 122        0.29722

 EXCITED STATE   7:      SINGLET-A      4.4986 EV  275.61 NM  F=0.0214  <S**2>=0.000
   114 -> 119        0.16724
   115 -> 119        0.46780
   116 -> 119       -0.40366
   117 -> 120       -0.13962

 EXCITED STATE   8:      SINGLET-A      4.7860 EV  259.05 NM  F=0.0340  <S**2>=0.000
   115 -> 120       -0.10077
   116 -> 119        0.23848
   116 -> 120       -0.11982
   117 -> 120       -0.15133
   117 -> 121        0.14766
   118 -> 120        0.35538
   118 -> 121       -0.32397
   118 -> 131        0.10439
   118 -> 132        0.10261
   118 -> 134       -0.11854
```

TD-DFT data of chromophore 11b

Excitation energies and oscillator strengths

```
 iMO      E(MO)   Type MO
  113    -0.31308   HOMO-4
  114    -0.30699   HOMO-3
  115    -0.29388   HOMO-2
  116    -0.27437   HOMO-1
  117    -0.25928   HOMO
  118    -0.04585   LUMO
  119    -0.00891   LUMO+1
  120    -0.00397   LUMO+2
  121    -0.00064   LUMO+3
  122    0.01216   LUMO+4

 EXCITED STATE   1:      SINGLET-A      3.8350 EV  323.30 NM  F=0.0197  <S**2>=0.000
   110 -> 118       -0.18364
   111 -> 118        0.61947
   111 -> 119        0.10067
   112 -> 118       -0.10206
   115 -> 118       -0.13980
 THIS STATE FOR OPTIMIZATION AND/OR SECOND-ORDER CORRECTION.
 TOTAL ENERGY, E(TD-HF/TD-KS) =  -1395.85385393
 COPYING THE EXCITED STATE DENSITY FOR THIS STATE AS THE 1-PARTICLE RHOCI DENSITY.

 EXCITED STATE   2:      SINGLET-A      4.0901 EV  303.13 NM  F=0.4596  <S**2>=0.000
   111 -> 118        0.14283
   113 -> 118        0.14468
   115 -> 118        0.50620
```

```
   117 -> 118       -0.37112

EXCITED STATE   3:      SINGLET-A      4.2543 EV  291.43 NM  F=0.0740  <S**2>=0.000
   116 -> 130       -0.17385
   117 -> 120        0.65565

EXCITED STATE   4:      SINGLET-A      4.4115 EV  281.05 NM  F=0.1756  <S**2>=0.000
   113 -> 118        0.10579
   114 -> 118       -0.29623
   115 -> 118        0.29715
   117 -> 118        0.42563
   117 -> 119       -0.18403
   117 -> 121        0.16683
   117 -> 123       -0.10757

EXCITED STATE   5:      SINGLET-A      4.4825 EV  276.60 NM  F=0.0650  <S**2>=0.000
   113 -> 118        0.46421
   114 -> 118        0.43915
   115 -> 119        0.13959
   117 -> 119       -0.10070

EXCITED STATE   6:      SINGLET-A      4.6550 EV  266.35 NM  F=0.2920  <S**2>=0.000
   116 -> 120        0.63619
   117 -> 130        0.16368

EXCITED STATE   7:      SINGLET-A      4.7097 EV  263.25 NM  F=0.1493  <S**2>=0.000
   113 -> 118        0.13855
   116 -> 120       -0.13782
   117 -> 118        0.23605
   117 -> 119        0.36895
   117 -> 121       -0.35716
   117 -> 123       -0.17716

EXCITED STATE   8:      SINGLET-A      4.8878 EV  253.66 NM  F=0.0506  <S**2>=0.000
   108 -> 118        0.12308
   113 -> 118       -0.26810
   113 -> 119        0.16177
   114 -> 118        0.33406
   114 -> 119       -0.11940
   115 -> 118        0.22785
   115 -> 119       -0.15178
   115 -> 121       -0.17105
   115 -> 126       -0.10769
   117 -> 118        0.18894
   117 -> 123       -0.10077
```
